# Phylogeny explains capture mortality of sharks and rays in pelagic longline fisheries: a global meta-analytic synthesis

**DOI:** 10.1038/s41598-022-21976-w

**Published:** 2022-10-28

**Authors:** Eric Gilman, Milani Chaloupka, Lee R. Benaka, Heather Bowlby, Mark Fitchett, Michel Kaiser, Michael Musyl

**Affiliations:** 1 The Safina Center, Honolulu, USA; 2 The Lyell Centre, Heriot-Watt University, Edinburgh, UK; 3 Ecological Modelling Services Pty Ltd and Marine Spatial Ecology Lab, University of Queensland, Brisbane, Australia; 4 Office of Science and Technology, U.S. NOAA Fisheries, Silver Spring, USA; 5 Bedford Institute of Oceanography, Fisheries and Oceans, Dartmouth, Canada; 6 Western Pacific Regional Fishery Management Council, Honolulu, USA; 7 Pelagic Research Group, Honolulu, USA

**Keywords:** Ecology, Environmental sciences, Ocean sciences

## Abstract

Apex and mesopredators such as elasmobranchs are important for maintaining ocean health and are the focus of conservation efforts to mitigate exposure to fishing and other anthropogenic hazards. Quantifying fishing mortality components such as at-vessel mortality (AVM) is necessary for effective bycatch management. We assembled a database for 61 elasmobranch species and conducted a global meta-synthesis to estimate pelagic longline AVM rates. Evolutionary history was a significant predictor of AVM, accounting for up to 13% of variance in Bayesian phylogenetic meta-regression models for Lamniformes and Carcharhiniformes clades. Phylogenetically related species may have a high degree of shared traits that explain AVM. Model-estimated posterior mean AVM rates ranged from 5% (95% HDI 0.1%–16%) for pelagic stingrays and 76% (95% HDI 49%–90%) for salmon sharks. Measures that reduce catch, and hence AVM levels, such as input controls, bycatch quotas and gear technology to increase selectivity are appropriate for species with higher AVM rates. In addition to reducing catchability, handling-and-release practices and interventions such as retention bans in shark sanctuaries and bans on shark finning and trade hold promise for species with lower AVM rates. Robust, and where applicable, phylogenetically-adjusted elasmobranch AVM rates are essential for evidence-informed bycatch policy.

## Introduction

Elasmobranchs (sharks and rays) belong to one of the most diverse marine taxonomic groups and include apex and mesopredators essential for maintaining ecosystem structure, functions and stability^1–3^. Overexploitation is the primary cause of declines of marine species. It can cause protracted or irreparable harm and permanent loss of populations, with changes and loss in marine biodiversity and ecosystem services^4,5^. Bycatch in pelagic longline fisheries is a global threat to the conservation of some elasmobranchs^6^. Pelagic sharks experienced a 71% decline in abundance over the past 50 years^7^. Depending on a fishery’s management framework and markets, catch composition and practices of individual vessels, sharks and rays may be discarded or retained as either incidental or target catch^8,9^. Retention may entail the entire fish or only shark fins or ray gill rakers.

There has been increasing concern in recent decades over the sustainability of elasmobranch mortality in pelagic fisheries given elasmobranchs’ vulnerability to exploitation, ecosystem-level cascading effects from declines in elasmobranch abundance, and fisheries-induced evolution and reduced population fitness that results from selective removals based on heritable traits^2,3,10–12^. There has also been increasing attention to the socioeconomic costs to fisheries from shark interactions such as from the depredation of catch and bait^8,13^ and risks to food, nutrition and livelihood security of coastal fishing communities from declining elasmobranch abundance^14,15^.

Accurate estimates of all sources of mortality are needed for robust assessments of the ecological effects of fishing, including through quantitative stock assessments, population viability models and multispecies ecosystem models^16,17^. For non-retained catch, a key component of total fishing morality is at-vessel mortality (AVM), also referred to as haulback or capture mortality, which is the proportion of the catch that is dead upon retrieval of the fishing gear before being handled by crew. Because small changes in mortality can result in large changes in mature biomass and therefore population growth, uncertainty in AVM rates is a major impediment to effective management and sustainable fisheries, in particular for elasmobranchs^18^. Robust estimates of species-specific AVM rates enable performance assessments of conservation and management measures, such as retention bans—including within Shark Sanctuaries, bans on international trade, bycatch quotas, shark finning bans, gear design requirements and handling-and-release practices. Identifying informative predictors of AVM risk, including phylogeny, physiological attributes, fishing methods and gear designs, facilitates estimation of AVM rates for data-limited species and identification of effective methods to mitigate AVM.

There is limited information available on AVM rates for most elasmobranchs, and quantitative meta-analytic synthesis studies of AVM rates in longline fisheries have been conducted for few elasmobranch species^18–21^. Independent and unbiased synthesis of all accumulated scientific information is a fundamental transparency principle for developing evidence-informed conservation management decisions^22,23^. Meta-analytic based synthesis approaches typically produce the strongest evidence with generalizable results that are optimal for global and regional decision-making^24,25^.

This study fills this priority research gap on elasmobranch AVM rates. We assembled an extensive database and conducted a global meta-synthesis to aggregate, test and summarize species-specific AVM rates for elasmobranchs captured in pelagic longline fisheries. The study significantly extends previous meta-analyses^26–30^ through: (1) the inclusion of a much larger number of studies and species; (2) applying Bayesian multilevel or hierarchical meta-regression models to account for informative covariates; and (3) phylogenetically adjusting the AVM rate estimates for the evolutionary history for two major shark clades with either relatively high (Carcharhiniformes) or low (Lamniformes) extant species diversity^31^. The study objectives were to:

1. Derive robust species-specific estimates and measures of uncertainty of AVM rates for elasmobranchs captured in pelagic longline fisheries;
2. Determine if phylogeny, operational longline fishing methods and gear designs, ventilation mode, morphology and ocean region are informative predictors of AVM rates; and
3. Identify gaps in information on priority potentially informative predictors and in taxonomic groups and regions for which additional primary studies are required for robust meta-syntheses of AVM rates.

The AVM rates derived for 61 elasmobranch species, estimated using robust statistical procedures for synthesizing evidence from multiple studies, supports evidence-informed conservation and fisheries management for sharks and rays.

## Results

### Predictor screening

The random forest derived variable importance plot identified several potentially informative predictors of elasmobranch AVM for the entire sample of 1,438 effect sizes summarized in Fig. S1. Only six predictors (genus, family, body type, number of hooks between floats, order, ocean) were deemed sufficiently informative with variable importance > 10%, which was used as the cut-off threshold. Several of those six predictors (genus, family, order) are taxonomic specific and accounted for most of the expected predictor importance but can be explicitly modelled using a single phylogenetic tree structure instead. We also included in our modelling workflow two less informative predictors shown in the variable importance plot (hook type, soak duration) as they are of general interest to fisheries managers because they are operational parameters that can be managed. Hence, all subsequent meta-analytic regression models considered this set of predictors in the Bayesian GAMMs.

### Model evaluation diagnostics

Convergence diagnostics such as multiple chain rank plots, and the effective posterior sample size (ESS) metrics coupled with the rank-based diagnostic statistic *Rhat* < 1.01^32^, reflected convergence of the Bayesian GAMMs with binomial-Normal likelihood. The best-fit GAMMs identified by the LOOcv and Bayesian stacking metrics fitted the clade-specific AVM datasets well as shown for example by the graphical posterior predictive check tests summarized in Fig. S2.1 for the model fitted to the 466 AVM study-specific effect sizes for 13 Lamniform species caught in pelagic longline fisheries operating in two ocean basins (Atlantic, Pacific).

### Clade-specific AVM estimates adjusting for phylogenetic structure

**Figure 1:**
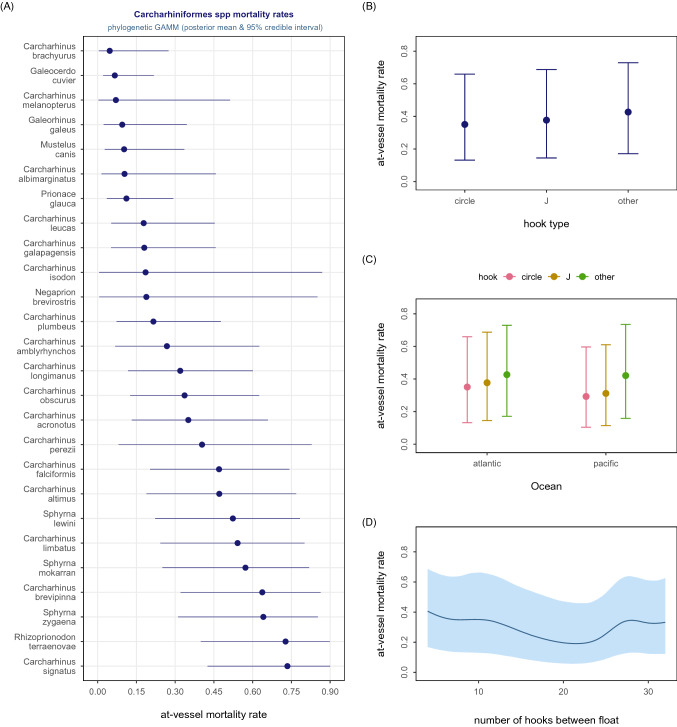
CARCHARHINIFORMES. Graphical summary of the Bayesian phylogenetic meta-regression model (binomial-Normal GAMM) fitted to 706 AVM effect sizes compiled for this 26 shark species clade. Panel (**A**) shows the estimated conditional species-specific AVM rate arranged from lowest to highest rate. Panel (**B**) shows the estimated conditional hook type effect on AVM. Panel (**C**) shows the estimated conditional effect of hook shape within ocean basin effect on AVM. Panel (**D**) shows the estimated conditional effect of number of hooks between floats on AVM. Solid dot = posterior mean, horizontal or vertical bar = 95% credible interval, solid curve = mean nonlinear trend, shaded polygon = 95% pointwise credible interval.

**Figure 2:**
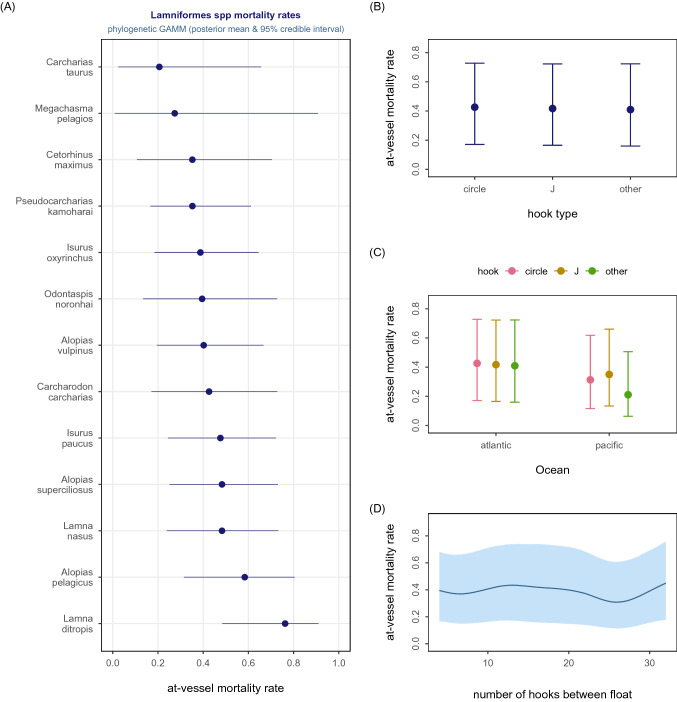
LAMNIFORMES. Graphical summary of the Bayesian phylogenetic meta-regression model (binomial-Normal GAMM) fitted to 466 AVM effect sizes compiled for this 13 shark species clade. Panel (**A**) shows the estimated conditional species-specific AVM rate arranged from lowest to highest rate. Panel (**B**) shows the estimated conditional hook shape effect on AVM. Panel (**C**) shows the estimated conditional effect of hook type within ocean basin effect on AVM. Panel (**D**) shows the estimated conditional effect of number of hooks between floats on AVM. Solid dot = posterior mean, horizontal or vertical bar = 95% credible interval, solid curve = mean nonlinear trend, shaded polygon = 95% pointwise credible interval.

The pooled mean AVM estimates for the Carcharhiniform species (Fig. 1A) and Lamniform species (Fig. 2A) were 0.376 [95% highest posterior density interval (HDI) 0.13–0.63] and 0.383 (95% HDI 0.15–0.65), respectively. In both models, only ocean basin (Figs. 1C, 2C) was a significant predictor of species-specific AVM rates in addition to the phylogenetic structure—hook type and number of hooks between floats were not significant predictors in either model (Figs. 1B,D, 2B,D).

### Phylogenetic signal

The mean phylogenetic signal or constraint estimated for Carcharhiniform species was estimated to be 0.06 (95% HDI 0.01–0.23) and 0.13 (95% HDI 0.01–0.42) for Lamniform species. These are significant but moderate phylogenetic signals, implying that evolutionary history played an important but relatively minor role in explaining the AVM rate differences among the 26 Carcharhiniform species and the 13 Lamniform species.

### Estimated ocean basin effect in phylogenetic models

Carcharhiniform species had a > 0.95 probability of lower AVM rates in the Pacific Ocean than the Atlantic Ocean. Carcharhiniform species in the Atlantic were 2.9 times (95% HDI 1.6–4.8) more likely to be dead at haulback than in the Pacific. The ocean-specific effect was significant for sharks caught on either circle or J-shaped hooks (Fig. 1C). A similar ocean-specific effect was evident for the Lamniformes (Fig. 2C) with the estimated mean marginal effect suggesting that Lamniform species caught in the Atlantic were 1.4 times (95% HDI 1.1–1.8) more likely to be dead at haulback than in the Pacific (Fig. S2.2).

### Publication bias

We found no evidence of potential publication bias for our phylogenetically adjusted meta-regression models that could be identified by funnel plot asymmetry based on a random-effects meta-regression model estimated within a frequentist inference framework. For instance, Fig. S3 shows a standard error-based contour-enhanced funnel plot for the Carcharhiniform species with little evidence for potential publication bias for this large data set of 706 study-specific effect sizes.

### AVM rate estimates from species-specific GAMMs

**Figure 3:**
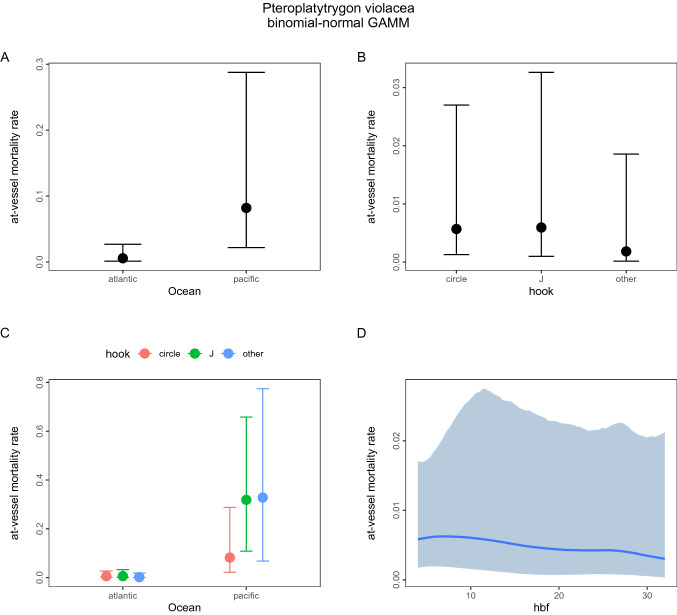
PELAGIC STINGRAY. Graphical summary of the Bayesian meta-regression model (binomial-Normal GAMM) fitted to 90 AVM effect sizes compiled for the pelagic stingray. Panel (**A**) shows the estimated conditional ocean-specific AVM rate. Panel (**B**) shows the estimated conditional hook shape effect on AVM. Panel (**C**) shows the estimated conditional effect of hook shape within ocean basin effect on AVM. Panel (**D**) shows the estimated conditional effect of number of hooks between floats on AVM. Solid dot = posterior mean, vertical bar = 95% credible interval, solid curve = mean nonlinear trend, shaded polygon = 95% pointwise credible interval.

The pelagic stingray *Pteroplatytrygon violacea* contained the largest number of records of the seven ray species in the assembled database (101 of 114). The overall AVM rate from a binomial-Normal GAMM was mean of 0.047 (95% HDI 0.001–0.157) (Table S1). Ocean basin (Fig. 3A) and hook shape in the Pacific Ocean (Fig. 3C) were significant predictors of pelagic stingray AVM. In combined ocean basins, hook shape (Fig. 3B) and number of hooks between floats (Fig. 3D) were not significant predictors. With > 99% certainty, the estimated AVM rate was significantly higher for the Pacific than the Atlantic Ocean effect sizes. The predicted marginal mean ocean-specific AVM rates, weighted proportionally according to sample size, for the pelagic stingray were 0.005 (95% HDI 0.001–0.02) for the Atlantic and 0.18 (95% HDI 0.04–0.41) for the Pacific Ocean effect sizes. In the Pacific, the pelagic stingray predicted marginal mean AVM rate for circle hooks was 0.08 (95% HDI 0.001–0.23) and 0.31 (95% HDI 0.001–0.61) for J-shaped hooks.

For giant manta ray *Mobula birostris*, cookie cutter shark *Isistius brasiliensis* and velvet dogfish *Zameus squamulosus*, overall AVM rates from binomial-Normal GAMMs were a mean of 0.332 (95% HDI 0.015–0.707), 0.295 (95% HDI 0.055–0.564), and 0.226 (95% HDI 0.071–0.368), respectively (Table S1). Only hook shape was a significant predictor of giant manta ray AVM rate. We can be > 96% sure that the estimated giant manta ray AVM rate was significantly higher on J-shaped than on circle hooks—the predicted marginal mean AVM rate for circle hooks was 0.19 (95% HDI 0.001–0.52) and 0.56 (95% HDI 0.01–0.99) for J-shaped hooks.

### Summary of species-specific AVM estimates

AVM rates were estimated for 54 shark species (n = 1322 records), and for 7 ray species (n = 116 records) (Table S1). Of these 61 species, 39 are in the two phylogenetic GAMMs. For sharks, AVM rates ranged from a low of a mean of 0.039 (95% HDI 0.01–0.07) for the picked dogfish *Squalus acanthias* to a high of a mean of 0.76 (95% HDI 0.49–0.90) for the night shark *Carcharhinus signatus*. For rays, mean AVM rates ranged from a low for the pelagic stingray (“AVM rate estimates from species-specific GAMMs” section) to a high of 0.5 (95% HDI 0.18–0.82) for the blackchin guitarfish *Glaucostegus cemiculus*.

Figure S4 is an example of a forest plot summarizing the model-predicted log relative risk ratios and the estimated random or Pooled Effects for the Atlantic sharpnose shark *Rhizoprionodon terraenovae*, with separate forest plots from records using only squid bait shown in Panel A and records using only fish, a mix of fish and squid, or unknown bait type in Panel B. Weighted relative risk estimates are ordered by effect size, and an estimated overall Pooled Effect log relative risk ratio is shown at the bottom of the forest plot and by the dashed vertical line. The overall AVM rate with only squid bait of 0.878 (95% HDI 0.75–0.99) was significantly higher than a mix of bait types of 0.427 (95% HDI 0.26–0.61).

## Discussion

### Phylogenetic signal

This is the first study to comprehensively assess the relationship between phylogeny and AVM risk for elasmobranch species exposed to pelagic longline fisheries. The finding of a moderate but significant relationship highlights the importance of accounting for phylogenetic dependence in elasmobranch multispecies meta-analytic syntheses on AVM rate^25,33,34^. Not accounting for phylogeny potentially risks estimating biased species-specific AVM rates, reduces the robustness of stock assessments, perhaps drawing incorrect conclusions and adopting misinformed policy. This finding is consistent with conclusions of previous studies that not accounting for phylogenetic non-independence of taxa in ecological meta-analyses produces biased results^33–35^.

More accurate estimates of shark species-specific AVM rates are obtained when meta-analytic syntheses are designed to account for phylogeny—the degree of shared evolutionary histories between species. Accounting for phylogeny in meta-analytic syntheses produces more robust species-specific AVM estimates for Lamniform and Carcharhiniform clades. The significant phylogenetic signal does not mean that species within these two clades have similar AVM rates; conversely, there is a broad range in species-specific AVM within these clades (Figs. 1, 2; Table S1). Instead, as with other significant explanatory predictors of AVM rates, accounting for phylogeny produces more accurate pooled estimates of species-specific AVM rates. Stock assessments, population models, multispecies ecosystem models, bycatch management strategy evaluations and other models that include AVM rates as a data input will in turn have more accurate findings, providing stronger evidence to guide decision-making.

The phylogenetic signal estimated by the Bayesian multilevel meta-regression models accounted for the dependence among elasmobranch species due to shared evolutionary histories. Closely related taxa share a phylogenetic history and would be expected to be ecologically similar^36^. They may have a high degree of shared non-heritable as well as heritable traits—if traits were conserved during evolution leading to descendent lineages, which occurs when traits are under strong selection (phylogenetic niche conservatism)^34,36–38^. For example, while there is some co-occurrence, most extant Lamniformes and Carcharhiniformes are ecologically differentiated by diet, morphology and in some cases habitat type^39^. Some shared physiological, morphological and behavioral traits may be informative predictors of AVM risk. For example, the lamnid sharks and common thresher shark (*Alopias vulpinus*) are the only elasmobranchs with regional red aerobic myotomal muscle endothermy^40,41^. They can occupy deeper and cooler habitats on an ephemeral basis. Not being dependent on ambient temperature for thermoregulation may contribute to their AVM rate being less affected by the duration hooked and ocean temperature relative to ectothermic elasmobranchs^42^. Endothermic sharks, which may have had a single origin in the Cretaceous, also tend to have larger body sizes than ectothermic sharks, which previous studies have found to be an informative predictor of AVM risk—larger fishes have larger energy stores relative to smaller individuals, making them more resilient to stressors, including a fight response to capture^43–46^. However, not all traits may be conserved during evolution. Trait divergence can occur during adaptive radiation, when species quickly diverge to avoid competition and fill different ecological niches^37^. Fisheries are also altering populations’ evolution through selective removals based on heritable traits^12^, possibly including through selective AVM within populations based on traits such as for fitness^47^.

Furthermore, it is likely that vulnerability to AVM is a function of both evolutionarily conserved and labile traits^48^. While conserved traits, such as thermal tolerance and niche tracking, will be expressed consistently by individuals of a population, flexible, labile traits such as stress response, shyness/boldness and timing of reproduction may have high variability in their expression both by an individual and between individuals of a population^48,49^. The strength of any phylogenetic signal may be decreased by this complex mixture of conserved and labile traits that affect AVM rates^49^.

### Population-level consequences and potential risk of extinction

Some sharks with high AVM rates have relatively high extinction risks due to pelagic longline fisheries. This includes the pelagic thresher shark (*Alopias pelagicus*), with a relatively high mean AVM rate of 58%, which is categorized as Endangered by IUCN^50^, with a relatively low phylogenetically adjusted *r*_max_ (maximum intrinsic rate of population increase, a standard measurement of population productivity and extinction risk) of 0.15^51^ and low mean *r*_max_ of 0.06^52^. Based on phylogenetically adjusted estimates of *r*_max_, Lamniformes have a high extinction risk and have moderate to high AVM rates. Carcarhiniformes had highest AVM rates but a lower extinction risk relative to Lamniformes^51^.

For individual fisheries with species-specific AVM rates that are substantially higher than the overall or pooled estimate for that species from the meta-analysis, modifications to operational explanatory predictors may reduce the AVM rate closer to the global pooled estimate. However, context-specific biological (e.g., size, sex) and environmental factors (e.g., thermocline depth, sea surface temperature, dissolved oxygen) may be more important predictors of AVM than manageable, operational fishing parameters. Similarly, there may be fisheries where a species tends to be retrieved alive but are subsequently discarded dead. For elasmobranch species with low AVM rates, in fisheries where these species have low retention, comparing at-vessel and release conditions would help determine if improved handling and release practices could increase the proportion of released catch that are alive, as well as reduce the post-release mortality rate. Furthermore, in fisheries where elasmobranch species with low AVM rates are retained, output controls that limit or ban their retention or trade have the potential to substantially reduce fishing mortality.

For elasmobranchs with relatively high AVM rates, methods that avoid and reduce their catch risk hold promise to reduce total fishing mortality. For these species, retention bans, shark finning restrictions and other output controls may be inappropriate approaches, unless they indirectly lead to the use of methods that reduce catch rates. Instead, measures that avoid and minimize shark and ray catch in pelagic longline fisheries are needed, either by: (1) reducing effort; or reducing one or more capture susceptibility attributes of: (2) spatial and temporal overlap through static and dynamic area-based management tools; (3) vertical overlap by managing fishing depth and the time of day of fishing; and (4) selectivity such as by adjusting leader material, hook and bait type and restricting the use of light attractors (Section S4). For species with relatively low AVM rates, in addition to catch avoidance and minimization approaches, prescribed handling and release practices (Section S5) may substantially reduce their total fishing mortality.

In addition, managing operational fishing methods and gear designs such as soak duration, fishing depth, branchline length, hook shape and size, bait type, leader material, time-of-day and fishing location can reduce elasmobranch AVM risk^18,53,54^. Supplemental Information Section S2 discusses the implications of findings for predictors of ocean basin, hook shape and the number of hooks between floats. Previous studies found that AVM rates for some individual elasmobranch species were a function of some of these operational predictors^27,53–56^. We found little support for such predictors affecting estimated AVM rates for our two clades of 39 shark species. Species-specific interactions with all operational predictors could not be explored for most of these 39 species because the publications compiled to assemble our dataset provided insufficient coverage of all predictors included in the models. However, the sample forest plot presented for the Atlantic sharpnose shark (Fig. S4) indicates that fisheries using only squid bait had a higher AVM rate than those not using only squid bait. This observed difference in AVM rates may have been caused by bait type. This observed effect conflicts with the prevailing understanding that, due to the prevalent hooking location, using squid, which results in a higher incidence of jaw hooking, instead of forage fish species for bait, which results in a higher incidence of gut hooking, might result in lower AVM rates^57^. Section S2.1 discusses a possible interacting effect of bait and hook type on anatomical hooking position. Bait type also affects size selectivity, an additional explanatory predictor of AVM^58,59^. Or it may have been due to various other differences between the two groups of fisheries. As with ocean basin (Section S2.1) and with hook shape (Section S2.2), there may have been simultaneous variability in other potentially significant predictors of AVM. However, for our single species GAMMs, we did have sufficient study-specific coverage of operational characteristics and could test whether such predictors were informative, with hook shape being a significant predictor in two of these models (Section S2.2).

Retention bans and limits, and prohibitions on shark finning and international trade might reduce shark and ray retention. This could substantially reduce the total fishing mortality of elasmobranchs with relatively low AVM rates and hence a relatively high capacity to be released alive. These measures, as well as bycatch quotas (a limit on the catch level of a bycatch species), may also cause fishers to discontinue using fishing methods and gear designs used to target sharks, such as shark lines, wire leaders, and using pieces of incidental catch for bait. If both a bycatch quota and consequences of reaching the threshold are sufficient, then quotas may incentivize fishers to adjust fishing gear and methods to increase selectivity^60–62^. Individual transferable bycatch quotas incentivize fishers to minimize their bycatch so that they can sell unused quota^61^. Fleetwide quotas, however, can incentivize a race for fish and can be inequitable as some vessels may be responsible for a disproportionate share of the quota-limited bycatch^63^. Robust monitoring systems are needed to produce accurate catch estimates of species subject to bycatch quotas. Because bycatch quotas increase the sensitivity of reporting bycatch data, observers are increasingly vulnerable to coercion, corruption and safety risks, which could be addressed by employing electronic monitoring systems^64,65^.

Bans on shark finning, where fins are retained and the remaining carcass is discarded, might reduce retention of sharks with little or no market value other than for the fins, and might discontinue fishing methods and gear designs used to target sharks. If shark carcasses have low market value, then shark finning bans will be a disincentive for targeting sharks, as fishers will want to use space in the fish hold for more valuable catch. However, for shark species that are retained for their meat and various other products, finning bans are unlikely to affect fishing mortality rates^9,66^. Furthermore, relative to species with low AVM rates, for shark species with high AVM rates, finning bans will be less effective at reducing fishing mortality.

Banning elasmobranch retention and international trade might cause pelagic longline fisheries to change targeting practices, discontinue retention and prevent sharks from becoming targets^67^. While most pelagic longline elasmobranch catch is from incidental capture by fisheries targeting tuna and tuna-like species (Scombroidei) and billfishes (Xiphioidei)^8,11^, these fisheries may also use gear designs to target sharks on shallower branchlines. This shark targeting would be discontinued if shark retention or trade were banned^68^ and subject to robust surveillance and enforcement. Several elasmobranch species are listed on Appendix II of the Convention on International Trade in Endangered Species of Wild Fauna and Flora, which establishes close control of their international trade, but none are currently listed on Appendix I, which would ban their international trade^69^. Retention bans, such as through measures adopted by regional fisheries management organizations (RFMOs) and under national Shark Sanctuaries, have been documented to decrease shark fishing mortality rates in some fisheries^68^ but may be ineffective under certain management frameworks^67,70^.

Reducing discards is prescribed in international guidelines and required by a growing number of fisheries management frameworks^71,72^. While discard bans may incentivize fishers to implement more selective fishing gear designs and methods to reduce catch rates of unwanted species and sizes of catch subject to the policy, this may be counterproductive, in particular for species with low AVM and post-release mortality rates^53^.

The five tuna RFMOs tap a very small subset of elasmobranch bycatch mitigation methods. They all ban shark finning and have retention bans in place for certain elasmobranch species. One has catch limits for two shark stocks, some restrict the use of wire leaders and shark lines, and some have voluntary guidelines on handling and release practices^73–77^. This leaves substantial opportunities to improve regional conservation and management measures for elasmobranch longline bycatch.

## Research priorities and conclusions

Completely resolving elasmobranch phylogeny may enable more robust accounting of phylogenetic dependence in multispecies meta-syntheses of AVM rates. Several species had insufficient sample sizes for robust meta-regression models. Additional haulback condition records are needed for these species, in particular for threatened, rare and phylogenetically distinct species which may play relatively large roles in shaping evolutionary processes and ecosystem resilience^2,78^. We found a paucity of elasmobranch haulback condition records from the Indian Ocean and Mediterranean Sea, and no records from the Black Sea. Addressing these regional gaps is an additional priority.

Richer datasets would enable the inclusion of additional potentially informative predictors of AVM. Numerous predictors could not be explored here due to limitations of the assembled database. This includes, for example, duration spent hooked (fight time), dissolved oxygen, sea surface temperature, depth of the thermocline/mixed layer, salinity, body size and sex of the catch, hook number of the catch (which hook between two floats and hence relative fishing depth), whether the catch was on a shark line, anatomical hooking position, hook size, hook degree of offset, and time-of-day of the gear soak^18,20,53,54,56^. Unfortunately, most of these variables are rarely or never measured.

Furthermore, many of the potentially informative predictors that were extracted from the compiled publications had low variability, including soak duration, hooks between floats and branchline length, the latter being particularly important for obligate ram-ventilating elasmobranchs^18^. Compiling records from a broader range of pelagic longline fisheries employing diverse fishing methods and gear designs, including from artisanal, small-scale longline fisheries, would enable a more robust assessment of the significance of these variables.

Phylogeny was a moderate but significant predictor of pelagic longline AVM rate. This was the first comprehensive study to assess the influence of elasmobranch phylogeny on AVM. Contrary to some hypotheses^20,53^, ventilation mode and body form (morphology) were not informative predictors of haulback condition for pelagic elasmobranchs in our study. Body form categories based on gill slit morphology and ventilation mode used here may be correlated with phylogeny^79,80^, so when models are phylogenetically adjusted, these variables become redundant.

The most promising approach to reduce total fishing mortality of species with high AVM rates is to employ methods that reduce catch risk. To reduce total fishing mortality of species with low AVM rates, in addition to reducing catchability, handling-and-release practices and policy interventions that, under certain enabling environments, reduce retention, including retention bans in blue water shark sanctuaries, and shark finning and trade bans, are appropriate. The elasmobranch AVM rates, estimated here through Bayesian meta-regression analysis, a robust statistical procedure for synthesizing evidence from multiple studies, support evidence-informed elasmobranch conservation and fisheries bycatch management. When combined with robust estimates of natural and other anthropogenic hazards, including other components of fishing mortality, these AVM rates can improve the reliability of models of elasmobranch population dynamics and support developing strategies to mitigate risks.

## Methods

### Data compilation

A two-tiered literature search was employed to compile relevant peer-reviewed published and grey literature and assemble a dataset suitable for meta-analytic evaluation. A link to the assembled dataset, which includes references for retained publications, is provided in the Data Availability section. The methods for the systematic literature review were adapted from the Reporting Standards for Systematic Evidence Syntheses^81^, Collaboration for Environmental Evidence^82,83^ and Preferred Reporting Items for Systematic reviews and Meta-Analyses^84^. An unstructured literature search was implemented by reviewing reference lists of the compiled publications from the systematic search. Supplemental Information Section S3.1 contains details on approaches employed for the literature review searches. The number of articles retrieved and screened and retained/discarded were recorded in a flow diagram (Supplemental Information Fig. S5).

### Statistical modelling approach

#### Datasets and model overview

Our dataset comprised 1438 study-specific summary or aggregate AVM rates for 61 elasmobranch species from 33 genera, 22 families and 9 orders. Two shark clades or taxonomic orders (Carcharhiniformes, Lamniformes) accounted for 39 of those species (ca 64%) and phylogenetic Bayesian meta-regression models were fitted to those data. Separate Bayesian meta-regression models without phylogenetic adjustment were fitted to the AVM data for 4 other species (2 Myliobatiformes spp, 2 Squaliformes spp)—far too few species to explicitly account for any phylogenetic resolution. Overall, we used some form of Bayesian meta-regression model to summarize the AVM rates for 43 of the 61 elasmobranch species (*ca.* 70%). We excluded from meta-regression modelling the remaining 18 species with limited data records, including zero dead for 1 or more of the records but, nonetheless, derived AVM rates for further qualitative consideration in our meta-synthesis. The AVM rate for each of those 18 species (15 genera, 14 families, 8 orders) was estimated using a Bayesian binomial likelihood estimator that accounts for zero recorded mortalities^85^. The mean posterior rate and a 95% highest posterior density interval (HDI) was summarized by sampling from a binomial likelihood with a Bayes-Laplace prior^85^ using the binom package for R^86^—rather than just using the raw study-specific summaries.

#### Predictor screening and missing predictor imputation

We extracted several AVM covariates or predictors for each study but including all predictors in our meta-synthesis workflow would increase the risk of model overfitting. So, we used the metaforest package for R^87^ to fit a random-effects weighted metaforest model with clustered bootstrap sampling to screen for potentially informative predictors using a variable importance metric. Briefly, metaforest implements a machine-learning based exploratory approach adapted from random forest algorithms^88^ to identify relevant linear or nonlinear predictors, and perhaps higher-order interactions, from a wide selection of predictors. Random forests are a commonly used machine-learning tool for classification and for ranking of candidate predictors based on variable importance measures^89^. We used those predictors identified using the variable important metric for all 1438 effect sizes in our subsequent Bayesian meta-regression modelling workflow that was based on a subset of those effect sizes. Some of the predictors such as soak duration of the gear and number-of-hooks-between-floats were incomplete with missing values ranging from 4% for hooks between floats and 14% for soak duration. So, we used the missRanger package for R^90^, which itself uses the ranger package for R^88^, to do fast missing value imputation by chained random forests. Here we also used predictive mean matching to avoid any imputation with values that were not present in the original data. This data set with imputed missing predictor values was then used for predictor screening for the entire data set of 1438 effect sizes.

#### Elasmobranch phylogenetic structure

The multilevel or hierarchical meta-regression modelling approach^91^ that we used accounted for elasmobranch species-level variance by including a phylogenetic correlation matrix derived from a phylogenetic tree. This matrix allowed us to account explicitly for correlated species-level random effects^33^. However, Chondrichthyan (shark, ray and chimaera) phylogeny remains unresolved^37,46,92–94^, so we derived the phylogenetic correlation matrix for all the 61 elasmobranch (shark and ray) species in our study by using the phylogenetic tree construction proposed by Stein et al.^95^ for 1192 Chondrichthyan species. Credible sets of the species-level phylogenetic history are available for subsetting and downloading at http://vertlife.org/phylosubsets. We recovered 100 random Chondrichthyan phylogenetic trees in NEXUS file format^96^ from a posterior of distribution of 10,000 phylogenetic trees using the “[fully resolved 1 fossil (set of 10 k trees)]” source—Upham et al.^97^ provide details on how these phylogenetic tree posterior distributions have been constructed using a Bayesian 2-level “backbone-and-patch” approach. We then randomly selected one tree from that 100-tree selection, which was used as the phylogenetic hypothesis or template for our meta-synthesis. Similar results were found using other randomly selected trees. With that phylogenetic tree we were then able to derive variance and correlation matrices using the ape package for R^98^ and tree visualisations using the ggtree package for R^99^—see Fig. S6 for a radial tree plot showing the proposed phylogenetic structure for the 61 elasmobranch species evaluated in our study. Clade-specific subsets of that tree (Carcharhiniformes, Lamniformes) and the derived correlation matrices were used for the phylogenetically adjusted Bayesian GAMM models fitted to the species-specific AVM rates for each clade.

#### Bayesian phylogenetic regression models

We modelled the effect of potentially informative predictors on elasmobranch species-specific AVM rates using a Bayesian multilevel or hierarchical regression modelling approach, which included a species-level phylogenetic structure as a group-level or random effect to adjust for any phylogenetic dependence^100^. We fitted this model to each of the two clades: the Carcharhiniformes (26 species, 8 genera, 3 families) and the Lamniformes (13 species, 9 genera, 6 families). These data comprised 706 study-specific effect sizes for the Carcharhiniformes and 466 effect sizes for the Lamniformes, covering both the Atlantic and Pacific Oceans for both clades—records from the Mediterranean and Indian Ocean were excluded from this meta-analysis due to small sample sizes. We fitted these meta-analytic models to the study-specific proportion for each species recorded dead at haulback in pelagic longline gear. The multilevel structure for each model comprised the individual study identification, multiple records nested within some studies and the research group cluster for the various studies (see Konstantopoulos^91^ for a discussion of multilevel meta-analytic models). We included the potentially informative covariates or predictors in the meta-regression structured models to evaluate whether AVM rate was a function of those predictors. We had previously screened a range of potential predictors to determine a minimal set of informative predictors using machine learning approaches discussed above.

We fitted these random-effects meta-regression models with binomial likelihood appropriate for proportion data^101^ using the Stan computation back-end^102^ via the brms interface for R^103^. This is also known as a binomial-Normal hierarchical meta-analytic model^104^ but was fit within a Bayesian inference framework^105^ using weakly informative priors^106^. It is a binomial-Normal GAMM regression model because: (1) the model likelihood is binomial to account for the proportion response data, (2) the group-level or random effects including the phylogenetic structure are sampled from a multivariate Gaussian distribution^104^, and (3) any nonlinear covariate functional form was accounted for using a thin plate regression spline basis commonly applied to fit generalized additive mixed models or GAMMs^107^. The fitted binomial-Normal model to the study-specific estimates for each species were then used to derive the overall or pooled mean AVM rate based on the posterior for each estimate comprising 10,000 samples or draws that were also used to derive the uncertainty estimates.

We used HDI as our measure of uncertainty, which is the shortest credible interval^108^. The HDIs were summarized from the posterior samples for each species meta-analytic model fit using the tidybayes package for R^109^. A probability statement about the existence of a particular predictor-specific effect can also be determined with those 10,000 draws using the probability of direction metric proposed recently using the BayestestR package for R^110^.

The estimated effect summaries based on the best-fit conditional regression GAMMs were then adjusted for variable sample size using the predicted estimated marginal means approach^111^ and implemented using the emmeans package for R^112^. We then summarized the marginal effect posterior densities to assess any apparent difference for instance between the estimated marginal AVM rates for the Atlantic and Pacific estimates. The posterior ratio summary was included in the summary plot for this effect.

We estimated the model-specific phylogenetic signal as the mean posterior proportion (with 95% HDI) of the summed group-level variances attributable to the phylogenetic group-level component—this approach assumes a Brownian diffusion or genetic drift process of character evolution for a continuous species-specific trait^113^. The phylogenetic signal or constraint is a simple metric that reflects the apparent effect of shared evolutionary history or ancestry on the specific trait or attribute state being evaluated^114^.

All models were fit with 4 Markov chains with 10,000 iterations per chain after a warm-up of 2000 iterations. Model convergence was assessed using parameter-specific diagnostics such as multiple chain rank plots, bulk and tail effective sample size metrics and a rank-based *Rhat* statistic^32^. We also used leave-one-out cross-validation (LOOcv) metrics and Bayesian stacking^115,116^ to explore any comparative difference in expected predictive accuracy between models fitted with or without a specific predictor such as for instance body form, ventilation mode or phylogenetic correlation structure. Further evaluation of the best-fit-model was then assessed using graphical posterior predictive checks^117^. All inference was then made using the best-fit model and the posterior predictive samples^118^.

#### Bayesian species-specific regression models

We modelled the effect of potential informative predictors on AVM rates for each of the following four species using a similar Bayesian GAMM modelling approach but without phylogenetic adjustment as no phylogenetic structure was relevant for this small subset of species—two Myliobatiformes species (*Manta (Mobula) birostris*, *Pteroplatytrygon violacea*) and two Squaliformes species (*Isistius brasiliensis*, *Zameus squamulosus*).

#### Publication bias

We explored potential publication bias^119^ for the best-fit clade-specific models using a standard error-based contour-enhanced funnel plot^120^, which was more readily implemented here within a frequentist rather than a Bayesian modelling framework. Specifically, we used predicted AVM rates for the Carcharhiniformes and Lamniformes clades estimated using the metafor package for R^121^ for fitting a clade-specific frequentist-based meta-analytic type model with the same species-level phylogenetic structure and the other group-level (or random) effects used in the Bayesian GAMMs. Metafor cannot directly fit a binomial-Normal GAMM (or GLMM) with phylogenetic structure, so we used the multivariate parameterization form of a meta-regression model in metafor^122^ with logit transformed AVM response to mimic a GLMM and specifically accommodate in metafor the more complex forms of random-effect structures used here including the phylogenetic tree structure^33^.

## Supplementary Information

Supplementary Information.

## Data Availability

The assembled database used for the meta-analyses is openly available from https://tinyurl.com/elasmobranch-mortality.
