# Supplementary material for: Phylogeny explains capture mortality of sharks and rays in pelagic longline fisheries: a global meta-analytic synthesis
Source: Sci Rep. 2022 Oct 28;12:18164. doi: 10.1038/s41598-022-21976-w (PMC9616952; doi:10.1038/s41598-022-21976-w)
Supplement: Supplementary file 1 — Supplementary Information. [file 41598_2022_21976_MOESM1_ESM.pdf]

Supporting Information

Phylogeny explains capture mortality of sharks and rays in pelagic longline fisheries: A global meta-analytic synthesis

Eric Gilman, Milani Chaloupka, Lee R. Benaka, Heather Bowlby, Mark Fitchett, Michel Kaiser, Michael Musyl

Contents

S1. RESULTS 2
S1.1. Predictor Screening 2
S1.2. Heterogeneity 3
S1.3. Lamniformes Phylogenetic Meta-Regression Model 4
S1.4. Publication Bias 6
S1.5. Summary of Elasmobranch Species-specific AVM Rate Estimates 7
S1.6. Sample Forest Plot 10
S2. DISCUSSION OF EXPLANATORY PREDICTORS AND INTERACTING EFFECTS OF COMPONENTS OF FISHING MORTALITY 12
S2.1. Ocean Basin 12
S2.2. Hook Shape 13
S2.3. Hooks between Floats – Index for Relative Fishing Depth 13
S2.4. Interacting Effects of Components of Fishing Mortality 14
S3. METHODS 15
S3.1. Systematic and Unstructured Literature Reviews 15
S3.2. Elasmobranch Phylogenetic Structure 20
S4. METHODS TO AVOID AND REDUCE ELASMOBRANCH CATCH IN PELAGIC LONGLINE FISHERIES 21
S5. PRESCRIBED ELASMOBRANCH HANDLING AND RELEASE PRACTICES AND EQUIPMENT FOR PELAGIC LONGLINE FISHERIES 21
S6. REFERENCES 23

S1. RESULTS

S1.1. Predictor Screening

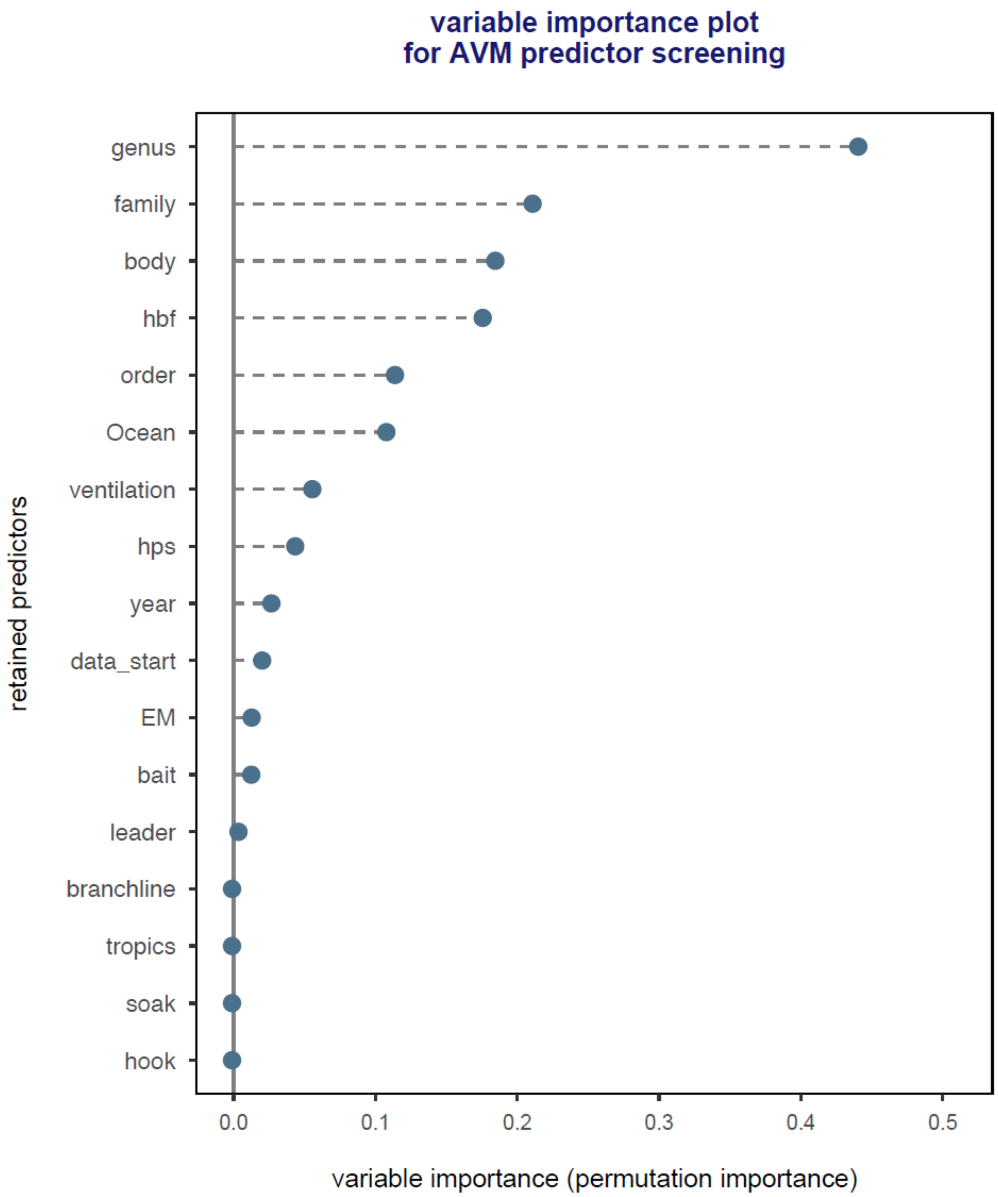

Fig. S1. Metaforest derived variable importance plot using chained random forests, identifying potentially informative moderator (or covariate) effects on at-vessel mortality (AVM) for the full dataset comprising 1,438 records for 61 elasmobranch species. [R^2*(oob)* = 0.034, R^2*(cv)* = 0.25, τ^2 = 1.29 = residual heterogeneity].

body = body type (morphology), 5 categories for sharks from Dolce and Wilga [74], and all rays; **hbf** = number of hooks between two floats; **Ocean** = Ocean basin; **ventilation** = respiratory mode of either obligate ram ventilator or buccal pump; **hps** = number of hooks deployed per set; **year** = year of publication; **data_start** = the first year of the time series; **EM** = data source (experiment, observer or electronic monitoring program, logbook program); **bait** = bait type (forage fish, squid, other); **leader** = leader material (wire, monofilament, other); **branchline** = branchline length; **tropics** = majority of fishing effort occurred in the tropics - between 23.4N and 23.4S (yes, no, other – mix or unknown); **soak** = maximum soak duration; **hook** = hook shape (circle, J-shaped J, tuna or teracima, other).

S1.2. Heterogeneity

Both Bayesian phylogenetic meta-regression models were good fits to the AVM data for either the Carcharhiniform or Lamniform species (Fig. S2.1). Nonetheless, there was significant between-study heterogeneity indicated by the following heterogeneity metrics [75] estimated for the Carcharhiniformes [median τ = 0.93 (95% HDI: 0.89-0.97); median I^2 = 99.8% (95% HDI: 99.7-99.9%)] and for the Lamniformes [median τ = 0.84 (95% HDI: 0.76-0.92); median I^2 = 98.2% (95% HDI: 96.1-99.3%)].

S1.3. Lamniformes Phylogenetic Meta-Regression Model

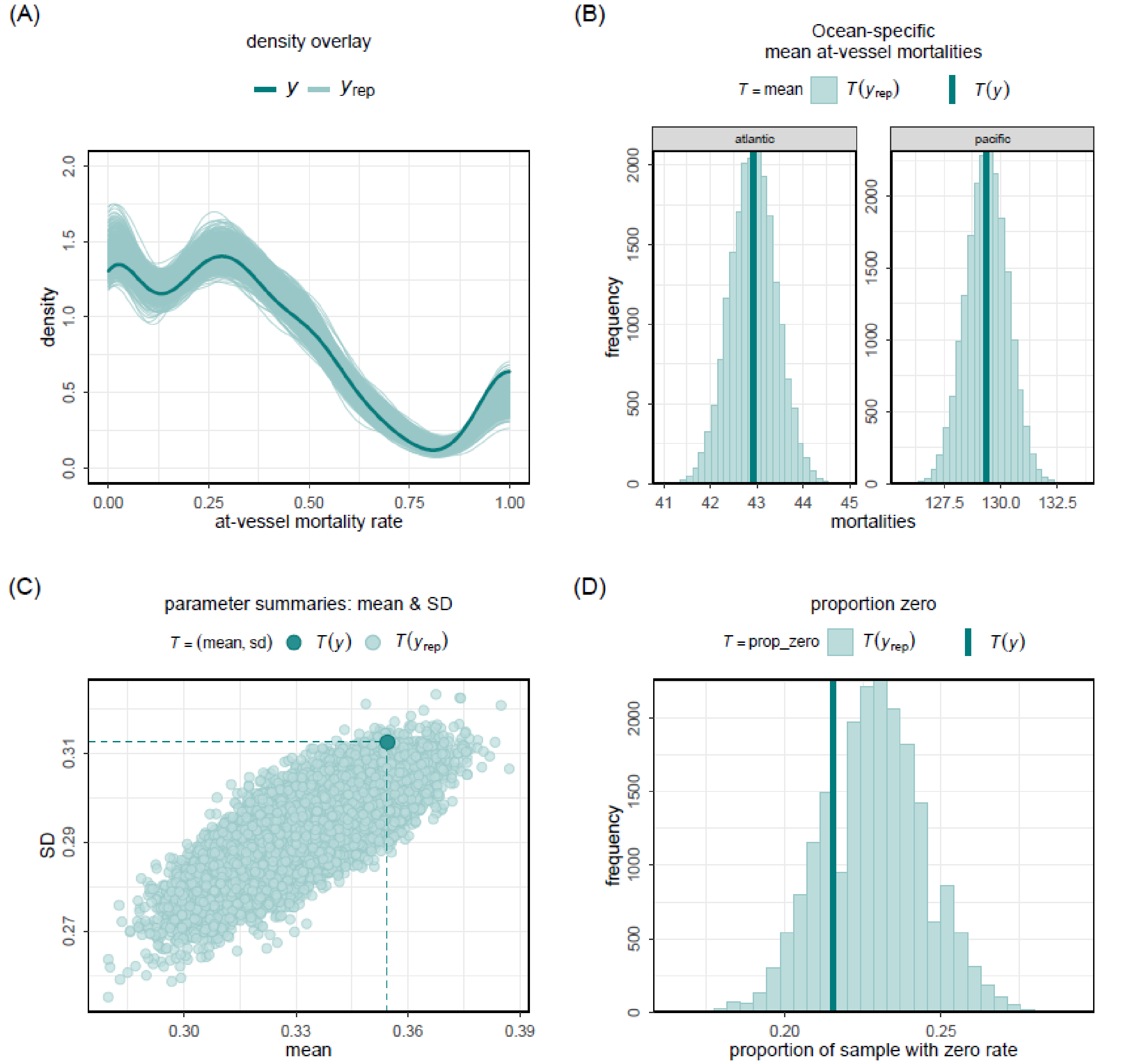

Fig. S2.1. Posterior predictive check tests for 1000 randomly selected draws from the best-fit phylogenetic binomial-Normal GAMM fitted to the AVM rates for the Lamniiformes species. (Panel A) shows the posterior predictive check for the response variable where the solid curve (y) is the density curve summarizing the observed rates while the mass of curves (y_{rep}) are 1000 model-based simulations of the expected post-release mortality rate. (Panel B) shows a group-specific check for the mean observed rate (solid vertical line) and the histogram of the expected rates (T(y)) conditional on whether deep- or shallow-hooked. (Panel C) shows a check for 2 key summary parameters (mean and standard deviation of the observed rate) where the solid dot = observed bivariate estimate and the mass of dots (T(y_{rep})) are the bivariate estimates for the 1000 model-based simulations. (Panel D) shows the observed proportion of zeroes (solid vertical line, T(y))) and expected proportion of zeroes for 1000 model-based simulations (light shaded vertical bars, T(y_{rep})). All check tests show that the best-fit model was an adequate fit to these data and therefore appropriate for inference.

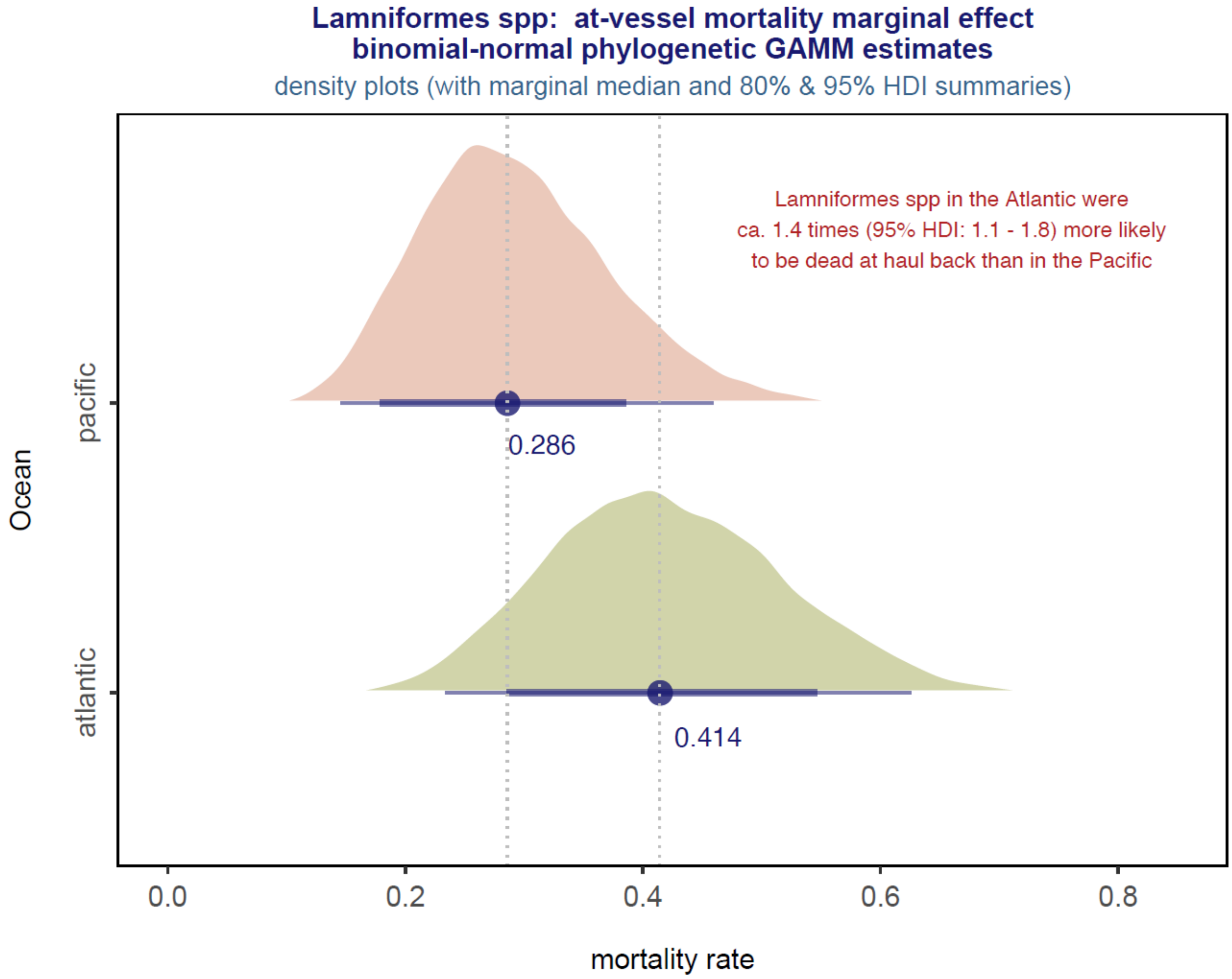

Fig. S2.2. Summary of the estimated marginal mean ocean-specific effect derived from the phylogenetic GAMM meta-regression model fitted to the Lamniformes AVM rates. Colored polygon shows the density distribution summary, solid dot (+ numeric label) = mean estimate of the density polygon, thick horizontal line below each polygon shows the 80% highest posterior density interval for the density polygon while the thin horizontal line is the 95% HDI.

S1.4. Publication Bias

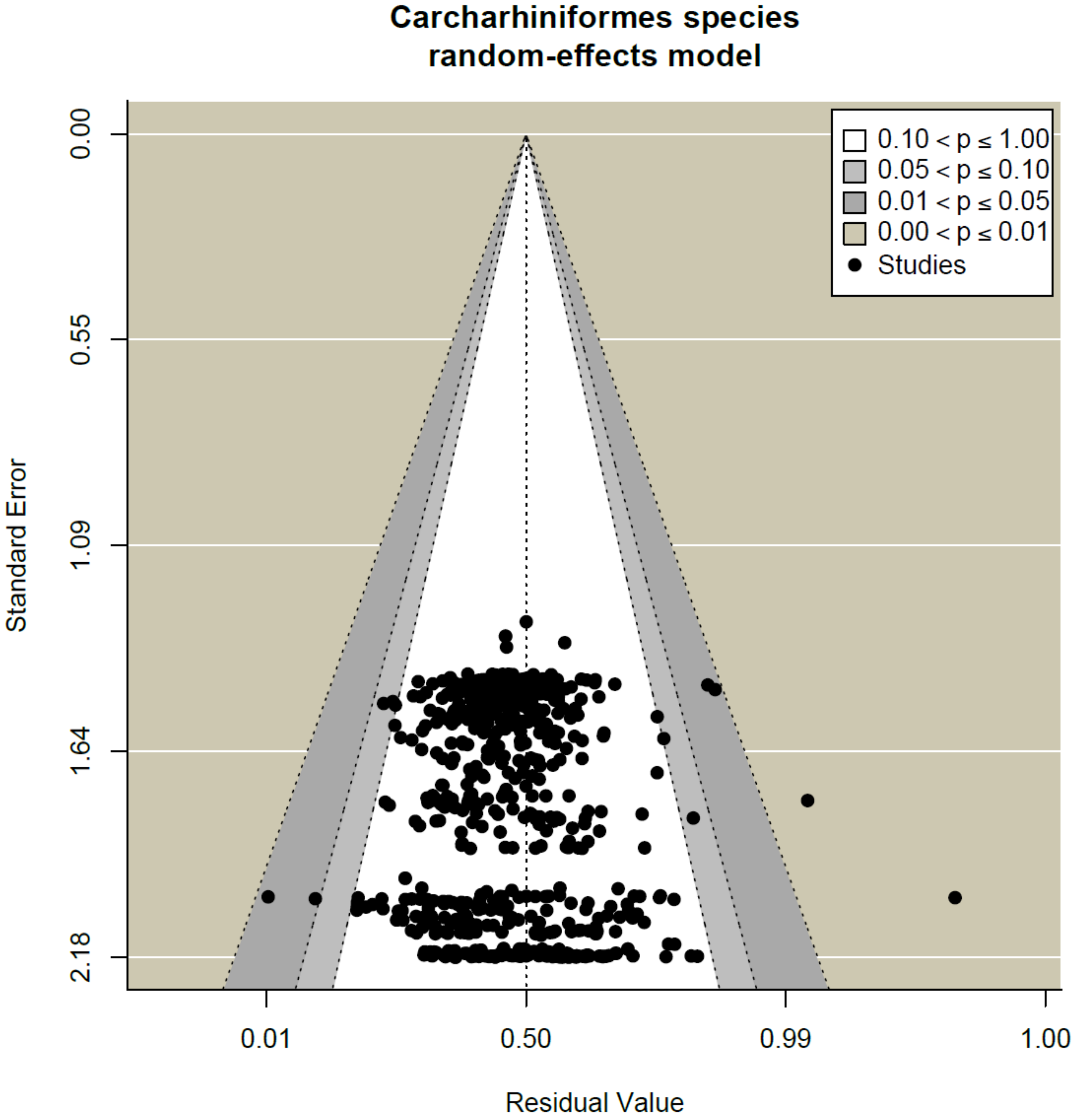

Fig. S3. Contour-enhanced funnel plot of the phylogenetically adjusted multivariate meta-regression model derived AVM estimates for the Carcharhiniformes study-specific effect sizes (N=706, 26 species).

S1.5. Summary of Elasmobranch Species-specific AVM Rate Estimates

Table S1. AVM rates for 61 elasmobranch species, identifying the modeling method used for each species-specific estimate. Records are arranged from highest to lowest posterior mean values.

		Species			posterior		95 % HDI	
**Order**	**Family**	**Scientific name**	**Common name**	**Method**	**median**	**mean**	**lower**	**upper**
Lamniformes	Lamnidae	Lamna ditropis	Salmon shark	phylogenetic GAMM	0.722	0.761	0.491	0.902
Carcharhiniformes	Carcharhinidae	Carcharhinus signatus	Night shark	phylogenetic GAMM	0.749	0.735	0.508	0.924
Carcharhiniformes	Carcharhinidae	Rhizoprionodon terraenovae	Atlantic sharpnose shark	phylogenetic GAMM	0.742	0.727	0.486	0.922
Carcharhiniformes	Sphyrnidae	Sphyrna zygaena	Smooth hammerhead shark	phylogenetic GAMM	0.659	0.641	0.380	0.864
Carcharhiniformes	Carcharhinidae	Carcharhinus brevipinna	Spinner shark	phylogenetic GAMM	0.654	0.637	0.388	0.881
Lamniformes	Alopiidae	Alopias pelagicus	Pelagic thresher shark	phylogenetic GAMM	0.530	0.584	0.304	0.754
Carcharhiniformes	Sphyrnidae	Sphyrna mokarran	Great hammerhead shark	phylogenetic GAMM	0.591	0.572	0.311	0.842
Carcharhiniformes	Carcharhinidae	Carcharhinus limbatus	Blacktip shark	phylogenetic GAMM	0.559	0.542	0.295	0.813
Carcharhiniformes	Sphyrnidae	Sphyrna lewini	Scalloped hammerhead shark	phylogenetic GAMM	0.542	0.524	0.270	0.789
Rhinobatiformes	Rhinobatidae	Glaucostegus cemiculus	Blackchin guitarfish	binomial (Bayes-Laplace prior)	0.500	0.500	0.180	0.820
Squaliformes	Centrophoridae	Centrophorus granulosus	Gulper shark	binomial (Bayes-Laplace prior)	0.500	0.500	0.150	0.850
Lamniformes	Lamnidae	Lamna nasus	Porbeagle shark	phylogenetic GAMM	0.432	0.485	0.208	0.654
Lamniformes	Alopiidae	Alopias superciliosus	Bigeye thresher shark	phylogenetic GAMM	0.430	0.484	0.214	0.650
Lamniformes	Lamnidae	Isurus paucus	Longfin mako shark	phylogenetic GAMM	0.425	0.477	0.215	0.650
Carcharhiniformes	Carcharhinidae	Carcharhinus altimus	Bignose shark	phylogenetic GAMM	0.489	0.471	0.224	0.764
Carcharhiniformes	Carcharhinidae	Carcharhinus falciformis	Silky shark	phylogenetic GAMM	0.487	0.470	0.230	0.734
Lamniformes	Lamnidae	Carcharodon carcharias	Great white shark	phylogenetic GAMM	0.375	0.428	0.142	0.648
Lamniformes	Alopiidae	Alopias vulpinus	Thresher shark	phylogenetic GAMM	0.354	0.406	0.168	0.578
Carcharhiniformes	Carcharhinidae	Carcharhinus perezii	Caribbean reef shark	phylogenetic GAMM	0.419	0.404	0.069	0.798
Lamniformes	Odontaspididae	Odontaspis noronhai	Bigeye sand tiger shark	phylogenetic GAMM	0.345	0.394	0.099	0.636
Lamniformes	Lamnidae	Isurus oxyrinchus	Shortfin mako shark	phylogenetic GAMM	0.340	0.390	0.151	0.557
Lamniformes	Cetorhinidae	Cetorhinus maximus	Basking shark	phylogenetic GAMM	0.307	0.356	0.072	0.608
Lamniformes	Pseudocarchariidae	Pseudocarcharias kamoharai	Crocodile shark	phylogenetic GAMM	0.305	0.354	0.130	0.511
Carcharhiniformes	Carcharhinidae	Carcharhinus acronotus	Blacknose shark	phylogenetic GAMM	0.367	0.351	0.133	0.628

		Species			posterior		95 % HDI	
Order	Family	Scientific name	Common name	Method	median	mean	lower	upper
Carcharhiniformes	Carcharhinidae	Carcharhinus obscurus	Dusky shark	phylogenetic GAMM	0.354	0.337	0.132	0.594
Carcharhiniformes	Carcharhinidae	Negaprion acutidens	Sicklefin lemon shark	binomial (Bayes-Laplace prior)	0.333	0.333	0.000	0.780
Hexanchiformes	Hexanchidae	Heptranchias perlo	Sharpnose sevengill shark	binomial (Bayes-Laplace prior)	0.333	0.333	0.030	0.670
Myliobatiformes	Mobulidae	Mobula japanica	Spinetail mobula	binomial (Bayes-Laplace prior)	0.333	0.333	0.000	0.780
Orectolobiformes	Rhincodontidae	Rhincodon typus	Whale shark	binomial (Bayes-Laplace prior)	0.333	0.333	0.000	0.780
Squatiniformes	Squatinidae	Squatina tergocellatoides	Ocellated angelshark	binomial (Bayes-Laplace prior)	0.333	0.333	0.000	0.780
Myliobatiformes	Mobulidae	Mobula birostris	Giant manta ray	GAMM	0.332	0.332	0.015	0.707
Carcharhiniformes	Carcharhinidae	Carcharhinus longimanus	Oceanic whitetip shark	phylogenetic GAMM	0.336	0.320	0.121	0.571
Squaliformes	Dalatiidae	Isistius brasiliensis	Cookie cutter shark	GAMM	0.278	0.294	0.055	0.564
Lamniformes	Megachasmidae	Megachasma pelagios	Megamouth shark	phylogenetic GAMM	0.232	0.273	0.000	0.818
Carcharhiniformes	Triakidae	Mustelus mustelus [1]	Smooth-hound shark	binomial (Bayes-Laplace prior)	0.268	0.268	0.156	0.384
Carcharhiniformes	Carcharhinidae	Carcharhinus amblyrhynchos	Grey reef shark	phylogenetic GAMM	0.281	0.268	0.058	0.586
Squaliformes	Centrophoridae	Centrophorus zeehaani	Southern dogfish	binomial (Bayes-Laplace prior)	0.250	0.250	0.000	0.630
Squaliformes	Somniosidae	Zameus squamulosus	Velvet dogfish shark	GAMM	0.225	0.226	0.071	0.368
Carcharhiniformes	Carcharhinidae	Carcharhinus plumbeus	Sandbar shark	phylogenetic GAMM	0.229	0.216	0.068	0.433
Lamniformes	Odontaspididae	Carcharias taurus	Sand tiger shark	phylogenetic GAMM	0.175	0.208	0.004	0.516
Hexanchiformes	Hexanchidae	Hexanchus griseus	Bluntnose sixgill shark	binomial (Bayes-Laplace prior)	0.200	0.200	0.000	0.530
Carcharhiniformes	Carcharhinidae	Negaprion brevirostris	Lemon shark	phylogenetic GAMM	0.198	0.188	0.000	0.783
Carcharhiniformes	Carcharhinidae	Carcharhinus isodon	Finetooth shark	phylogenetic GAMM	0.196	0.185	0.000	0.798
Carcharhiniformes	Carcharhinidae	Carcharhinus galapagensis	Galapagos shark	phylogenetic GAMM	0.191	0.180	0.038	0.398
Carcharhiniformes	Carcharhinidae	Carcharhinus leucas	Bull shark	phylogenetic GAMM	0.189	0.178	0.045	0.406
Squaliformes	Somniosidae	Centroscymnus owstonii [2]	Roughskin dogfish shark	binomial (Bayes-Laplace prior)	0.124	0.124	0.120	0.130
Carcharhiniformes	Carcharhinidae	Prionace glauca	Blue shark	phylogenetic GAMM	0.119	0.111	0.028	0.251
Carcharhiniformes	Carcharhinidae	Carcharhinus albimarginatus	Silvertip shark	phylogenetic GAMM	0.110	0.104	0.003	0.392
Carcharhiniformes	Triakidae	Mustelus canis	Dusky smooth-hound shark	phylogenetic GAMM	0.109	0.102	0.019	0.284
Rajiformes	Rajidae	Raja clavata	Thornback ray	binomial (Bayes-Laplace prior)	0.100	0.100	0.000	0.280
Carcharhiniformes	Triakidae	Galeorhinus galeus	Tope shark	phylogenetic GAMM	0.101	0.095	0.014	0.285

		Species			posterior		95 % HDI	
Order	Family	Scientific name	Common name	Method	median	mean	lower	upper
Myliobatiformes	Dasyatidae	Taeniura grabata	Round stingray	binomial (Bayes-Laplace prior)	0.083	0.083	0.000	0.240
Carcharhiniformes	Carcharhinidae	Carcharhinus melanopterus	Blacktip reef shark	phylogenetic GAMM	0.075	0.071	0.000	0.421
Carcharhiniformes	Carcharhinidae	Galeocerdo cuvier	Tiger shark	phylogenetic GAMM	0.071	0.066	0.016	0.175
Myliobatiformes	Mobulidae	Mobula mobular	Devil fish	binomial (Bayes-Laplace prior)	0.059	0.059	0.000	0.170
Myliobatiformes	Mobulidae	Mobula tarapacana	Chilean devil ray	binomial (Bayes-Laplace prior)	0.059	0.059	0.000	0.170
Myliobatiformes	Myliobatidae	Pteromylaeus bovinus	Bull ray	binomial (Bayes-Laplace prior)	0.056	0.056	0.000	0.160
Orectolobiformes	Ginglymostomatidae	Ginglymostoma cirratum	Nurse shark	binomial (Bayes-Laplace prior)	0.056	0.056	0.000	0.160
Carcharhiniformes	Carcharhinidae	Carcharhinus brachyurus	Copper shark	phylogenetic GAMM	0.050	0.047	0.001	0.215
Myliobatiformes	Dasyatidae	Pteroplatytrygon violacea [3]	Pelagic stingray	GAMM	0.019	0.047	0.001	0.157
Squaliformes	Squalidae	Squalus acanthias	Picked dogfish	binomial (Bayes-Laplace prior)	0.039	0.039	0.010	0.070

[1] The 7 compiled records for the common smooth-hound shark *Mustelus mustelus* were all from the Mediterranean Sea.

[2] The 2 compiled records for the roughskin dogfish shark (Owston's dogfish shark) *Centroscymnus owstonii* were both from the Pacific Ocean.

[3] The predicted marginal mean ocean-specific AVM rates, weighted proportionally according to sample size, for the pelagic stingray *Pteroplatytrygon violacea* were 0.005 (95% HDI: 0.001–0.02) for the Atlantic and 0.18 (95% HDI: 0.04–0.41) for the Pacific Ocean effect sizes. In the Pacific, the pelagic stingray predicted marginal mean AVM rate for circle hooks was 0.08 (95% HDI: 0.001-0.23) and 0.31 (95% HDI: 0.001-0.61) for J-shaped hooks.

S1.6. Sample Forest Plot

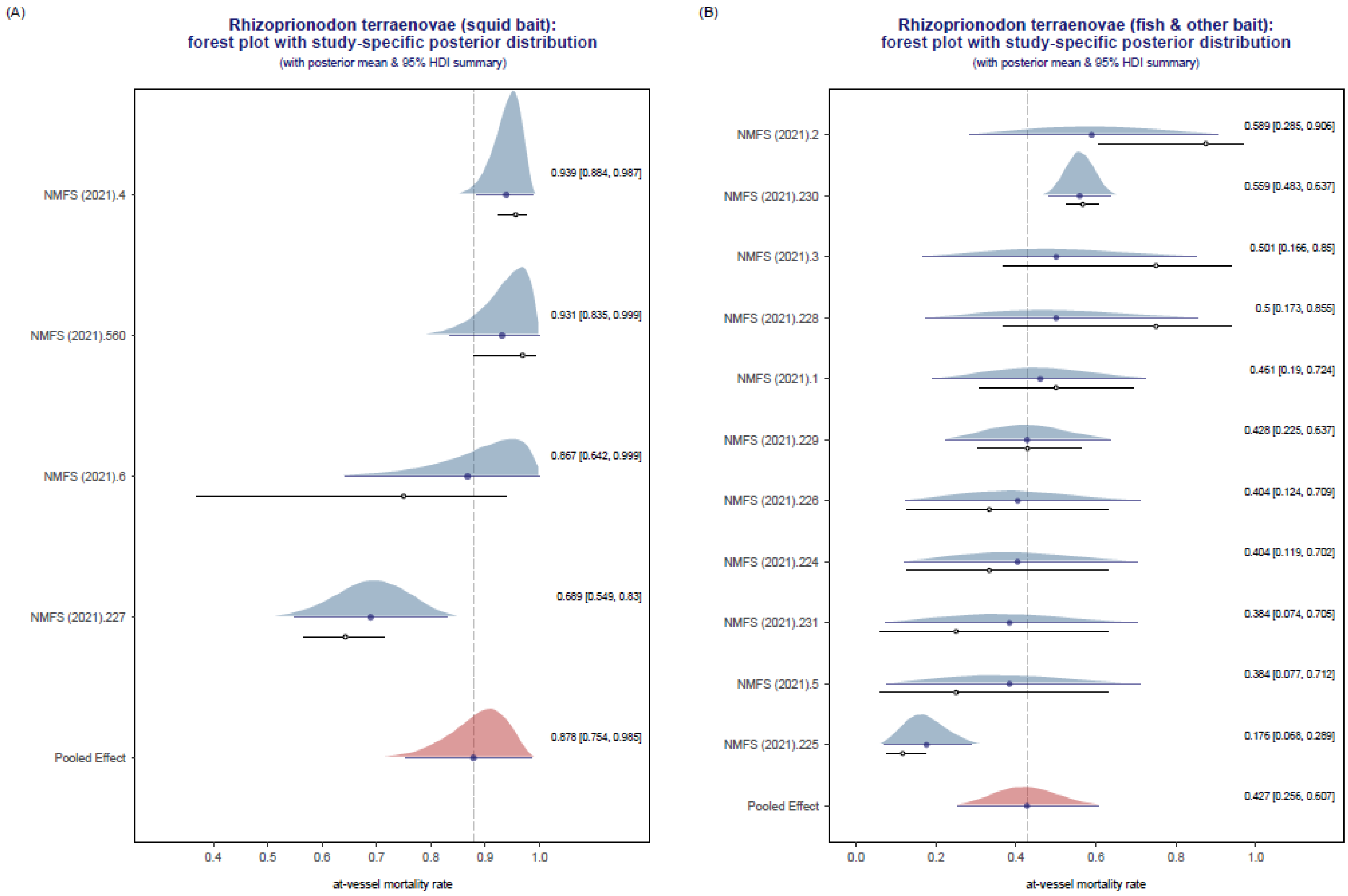

Fig. S4. Model-predicted log relative risk ratio derived for Atlantic sharpnose shark *Rhizoprionodon terraenovae* for 4 effect sizes for records using only squid for bait (Panel A) and 11 effect sizes for records using only fish, a mix of fish and squid, or unknown bait

type (Panel B). Shrinkage estimates were derived using a precision weighted Bayesian random-effects meta-analytic model with Gaussian likelihood. Polygon = density of the posterior draws (the effective sample size = 10,000) and reflects the distribution of each estimate, where a wide and thin polygon indicates low precision from a small sample size, and narrow and tall indicates there was high precision and large sample size. The horizontal line underneath each polygon = 95% HDI of the posterior draws, solid dot = mean of the posterior draws shrunk towards the random or Pooled Effect, which is the mean or overall expected log relative risk ratio (right-side labels = the posterior mean and HDI summaries). Below the density polygon is an open dot = observed effect size and thin horizontal line = observed effect size ± 1 standard deviation derived using the `metafor::escalc()` function. The difference between solid and open dots reflects the degree of shrinkage that is dependent on sample size.

S2. DISCUSSION OF EXPLANATORY PREDICTORS AND INTERACTING EFFECTS OF COMPONENTS OF FISHING MORTALITY

S2.1. Ocean Basin

There was a significant ocean basin effect on elasmobranch AVM rates. For the assembled database, shark AVM rates were generally higher in the Atlantic than the Pacific. The opposite effect occurred for pelagic stingray, the main ray species represented in the assembled database and captured in global pelagic longline fisheries [1].

This observed ocean basin effect could be due to biological (e.g., body size) or behavioral (e.g., fight response to capture) differences between populations of a species in different oceans, or because thermocline depth, temperature, dissolved oxygen, salinity and other environmental parameters that affect at-vessel condition vary over broad spatial and temporal scales ([2-5]. The basin effect could also have been due to correlations between ocean basin and various informative predictors of AVM. For example, Pfaller et al. [6] determined that an ocean effect on adult marine turtle survival rates was correlated with study methodology, including tag type, habitat type from which turtles were sampled, as well as statistical modeling approaches. In the current study, several environmental and operational (gear design and fishing method) informative predictors, and regional differences in species composition of the catch were likely correlated with ocean basin.

The species composition in the two regions was substantially different for the Carcharhiniformes model. The species with the five highest overall global AVM rates made up only 7.5% of the Pacific Ocean records but 21.5% of Atlantic Ocean records. In the Pacific Ocean, over 26% of Carcharhiniformes records were for the blue shark, which had the seventh lowest overall global AVM rate of the 26 species in the order included in the assembled database (Table S1). The distribution of Lamniformes records by species and global pooled AVM rates in the two regions was less variable than for the Carcharhiniformes. There were large differences in the latitudinal distribution of records by region, with *ca.* 75% of Pacific Ocean and *ca.* 25% of Atlantic Ocean Carcharhiniformes, Lamniformes and pelagic stingray records occurring in the tropics. A latitude effect may explain why regions within the Atlantic was found in a previous study to be an important predictor of species-specific AVM risk [7,8]. Latitude is a proxy for ocean temperature and dissolved oxygen, which are informative predictors of at-vessel condition due to species- and size-specific physiological stress responses and tolerance thresholds [4,5,9-13]. A latitude effect may also be due to the spatial segregation by size and sex within elasmobranch species [10,14]. Differences in survival probability have been observed by size and sex within species for some elasmobranchs [3,9,15-17]. A lower probability of AVM occurs with an increase in body size for some species [11,12,18,19]. Higher AVM rates for males than females have also been observed for some elasmobranch species [7,16,20]. This may be due to differences in body size by sex for species that exhibit sexual size dimorphism (differences in size by sex of the same age class, i.e., differential growth by sex). The effect of sex on AVM risk may also be due to differences in depth distributions by sex and concomitant differences in environmental explanatory predictors, and to physiological differences, such as different energy demands, and different skin thickness when females are reproductively active [3,10].

The ocean basin effect in the two phylogenetic and the pelagic stingray models may have also been due to large operational differences between the Atlantic and Pacific Ocean fleets. The use of different pelagic longline gear designs and fishing methods to target different species can significantly explain elasmobranch AVM rates [9]. Atlantic Ocean records in the three models were predominantly from shallow-set fisheries with about 5 hooks between floats, squid used for bait in about 30% of records, and wire leaders used in about 4% of records.

Elasmobranchs captured on shallow gear may be trapped in the uniform mix-layer and not be able to dive deeper to cool off and repay oxygen depts. Pacific Ocean records in the three models were largely from deep-set fisheries with about 19 hooks between floats, squid used for bait in about 12% of records, and wire leaders used in about 35% of records. A larger proportion of some sharks has been observed to be alive on wire than monofilament leaders [21,22]. Leader material may affect within-species selectivity by relative strength and vigor. While wire leaders tend to indiscriminately retain all sharks that ingest baited hooks, for sharks caught on monofilament leaders, stronger and more vigorous individuals may have a higher probability of escaping than weaker and more seriously injured individuals. Individuals retained on monofilament leaders may therefore have a higher probability of being dead upon haulback than sharks that are able to sever the monofilament leader and escape [21,22]. However, there may be synergistic effects of hook, bait and leader types on shark AVM rates. If hook and bait types are used that result in mouth hooking, then this may diminish or eliminate any effect of leader material on at-vessel condition [22]. As with hook shape (discussed below), bait type affects anatomical hooking position, which is an explanatory predictor of haulback condition, and can also affect size selectivity [7,23]. Due to the prevalent hooking location, using squid instead of forage fish species for bait might result in lower AVM rates [24]. This bait effect on anatomical hooking position may occur only when used in combination with J-shaped hooks [25]. Fishing depth is also an important predictor of haulback condition, discussed below. Hook type and mean maximum soak duration were similar in the two regions.

S2.2. Hook Shape

Pelagic stingrays had a 74% lower mean AVM rate on circle than J-shaped hooks from records in the Pacific Ocean. Similarly, giant manta rays had a 66% lower mean AVM rate on circle than on J-shaped hooks. Hook shape was not an informative predictor in the phylogenetic GAMMs (Figs. 1 and 2) nor in the predictor screening for the full dataset (Fig. S1). For most organisms that tend to be captured by ingesting a hook, circle hooks tend to lodge in the corner of the mouth while J-shaped hooks tend to result in deep hooking, lodging internally in the esophagus and gut [8,24,26,27]. Mouth and externally-hooked organisms have lower AVM rates relative to deeply hooked catch [24,28,29].

However, neither pelagic stingrays nor manta rays tend to be captured by ingesting a hook. Pelagic stingrays tend to be hooked in the mouth regardless of hook type [22,29,30], while filter-feeding mobulid rays may be captured predominantly by foul hooking in the body and entanglement in line [31]. The cause of the substantially lower pelagic stingray and giant manta ray AVM rates on circle relative to J-shaped hooks is therefore unclear. As with ocean basin, this could have been due to correlations between hook shape and other explanatory predictors of haulback condition. For example, pelagic stingray Pacific Ocean records using circle hooks used longer branchlines (mean of 13.9 m) than records using J-shaped hooks (mean of 12.5 m). For this obligate ram ventilator, the shorter branchlines may have been more likely to impair their movement during the gear soak, contributing to the higher AVM rate on J-shaped hooks [5,32]. Also, because some captured pelagic stingrays do ingest hooks deeply, if circle hooks had a wider minimum width than J-shaped hooks, because wider circle hooks are expected to result in a lower rate of deep-hooking relative to narrower J-shaped hooks [22], this correlation between hook shape and width may have contributed to the higher AVM rate on J-shaped hooks.

S2.3. Hooks between Floats – Index for Relative Fishing Depth

The number of pelagic longline hooks that are attached between two floats is an approximate index for relative fishing depth. The more hooks that are deployed between two floats, the deeper the depth range of the hooks along a catenary curve will be if all other variables are

constant. Differences in other factors that affect fishing depth, including shoaling from ocean currents and wind, and variability in other gear designs (e.g., length of mainline between floats, mainline diameter, distance between floats, distance between the point of attachment to the mainline of the first branchline and the point of attachment of the nearest floatline, distance between branchlines, and length of branchlines and floatlines) will determine the absolute depth range of the longline hooks [33-35].

There is variability in the degree of barotrauma, dissolved oxygen, salinity, temperature and other environmental variables with capture depth. These environmental variables affect physiology, metabolism, movement and stress, which are important predictors of at-vessel condition [3,5,9,16]. The effect of fishing depth on haulback condition will vary spatially and temporally due to variability in environmental conditions, including the depth of the mixed layer and oxygen/temperature gradients. In general, because shallower, warmer water generally has lower dissolved oxygen concentration, higher stress occurs for fish caught at shallower depths due to a combination of temperature, limited oxygen and higher metabolic rates [9,36]. However, the effect of capture depth on AVM risk is species-specific, and size-specific within species, as different species and sizes have different environmental tolerances [15]. For example, while carcharhinid sharks have higher AVM risk with higher water temperature and shallower capture depth (i.e., preventing vertical migration to deeper, cooler waters), epipelagic sharks exhibit the opposite response (i.e., an increased risk of AVM when captured below the mixed layer) [9,11,16]. Therefore, tradeoffs result from adjusting fishing depth, which will reduce AVM rates for some species, but increase rates for others [15,37].

S2.4. Interacting Effects of Components of Fishing Mortality

There are likely interacting effects between the components of fishing mortality. For example, relative to live catch, if dead catch is more likely to be depredated from the gear by scavengers, and thus is less likely to be retained on the gear by the haulback than live catch, then a higher pre-catch mortality rate would be associated with a lower AVM rate [38]. However, in particular for species with relatively low pre-catch mortality rates, a longer gear soak and other factors may result in higher escapement rates, also contributing to higher AVM rates. Which of these has a larger effect on retention is likely species- and fishery-specific, depending on, for instance, how strenuously the species attempts to escape, the local abundance of scavengers, effects of mechanical action, and gear designs (e.g., hook shape and leader material).

S3. METHODS

S3.1. Systematic and Unstructured Literature Reviews

Web of Science and Google Scholar databases were searched for the systematic literature review. The Web of Science proprietary database contains information on peer-reviewed articles from journals in the natural sciences, social sciences, arts and humanities, selected based on their meeting high editorial standards and scientific impact, with records from 1975 [39]. While Web of Science and similar bibliographic databases mainly include only established peer-reviewed journals, Google Scholar searches a wider range of sources, including conference proceedings, theses, books and book chapters, technical reports and various grey literature. Furthermore, for narrow literature searches, Google scholar, which searches the entire text, may identify relevant publications that are missed by main bibliographic databases, which only search keywords, abstracts and titles [40]. Two specialized databases were also searched to potentially identify unique references related to their specific narrow focuses that might not identified through Wed of Science or Google Scholar searches [40]. These were the Western and Central Pacific Fisheries Commission's Bycatch Management Information System (BMIS) online database of references (www.bmis-bycatch.org/references) and the Consortium for Wildlife Bycatch Reduction's online database of references (www.bycatch.org/search).

In order to be retained, a publication had to meet screening criteria of reporting, for one or more elasmobranch species, the number that was alive and number that was dead upon haulback (before being handled by crew) in pelagic longline gear. For the Web of Science and Google Scholar searches, we explored numerous alternative search strings of combinations of keywords (search terms) and Boolean operators designed to obtain publications that meet the screening criteria, and to restrict the combined number of results (hits) to ~1,300. The retained search strings had a specificity with ≥45% of 20 sampled titles of hits (first and last 10 titles for the combined hits from the Web of Science searches, and for the combined hits from the Google Scholar searches) being retained.

Because no publications are considered 'Gold Standard' for this research question, the sensitivity of the combined search string results was not assessed by determining inclusion of specific publications. Furthermore, relying on highly recognized publications to assess sensitivity, as recommended by CEE and ROSES [41-43], risks introducing prevailing paradigm and publication bias [44,45], where, for example, individual studies finding conflicting or non-significant results may not be highly cited, published or easily discoverable. The inclusion of grey literature and inclusion of all hits from database searches (and not for instance limiting results to the subset of the first 100 hits, which may be sorted by number of citations) contributed to reducing the risk of introducing these sources of bias.

The following four search strings were used for the Web of Science: (1) pelagic AND longline AND mortality; (2) "pelagic longline" AND (haulback OR at-vessel) AND mortality; (3) "pelagic longline" AND (haulback OR at-vessel) AND (mortality OR survival) AND shark -demersal; and (4) allintitle: "pelagic longline" AND (mortality OR survival). Four search strings using Google Scholar were: (1) "at-vessel" mortality shark "pelagic longline" fisheries; (2) haulback mortality shark "pelagic longline" fisheries -demersal -bottom; (3) mortality "blue shark" "pelagic longline" alive dead ray soak hook -demersal; and (4) allintitle: "pelagic longline" mortality OR survival -demersal -bottom. Searches without "allintitle" term searched for keywords anywhere in the text. The Google Scholar search was conducted without being affected by the browsing history, IP address and other personalization factors. The BMIS search was conducted for longline fishing gear and elasmobranchs, and keyword 'mortality', with no restrictions on year or collection category. The Consortium for Wildlife Bycatch Reduction database search was conducted for hooks-and-lines fishing gear and elasmobranchs, including both field and non-field studies, with no restrictions on mitigation technique. Searches of the four

databases were conducted on 2 October 2021. To reduce language bias and avoid temporal bias [43,45], searches did not restrict the time period or language of publications, but only English search terms were used. The number of articles retrieved, screened and retained/discarded from the systematic review were recorded in a flow diagram adapted from Haddaway et al. [46] (Fig. S5).

We then conducted an unstructured literature search by reviewing reference lists of the compiled publications from the systematic search (snowballing, [43]) and obtained primary observer program data, resulting in the retention of an additional 12 publications. We screened the compiled literature against the aforementioned eligibility criteria for retention. Both primary research and synthesis studies were considered for retention. Publications were not excluded based on a study design eligibility criterion. Published and grey literature results did not need to be peer reviewed in order to be retained. Of 64 retained publications from the systematic review, 16 were not included in an assembled database of records for use in meta-analytic models because they contained a subset of data from observer program datasets [47-50] that were compiled through the unstructured review. One of the retained systematic review publications is a corrigendum (containing a corrected table, [51]); only the original publication [21] is referenced in the final assembled database. A total of 59 publications were included in the final assembled database, of which 47 were derived from the systematic literature review and 12 from the unstructured review. Several variables were extracted from the compiled and retained publications, including several predictors of AVM risk defined in the caption of Fig. S1: number of hooks between floats, ocean basin, whether the majority of fishing effort occurred in the tropics, hooks per set, publication year, date of the start of the time series, data source, leader material, branchline length, maximum soak duration, and hook shape.

A Preferred Reporting Items for Systematic reviews and Meta-Analyses (PRISMA, [52,53] checklist follows.

Title: Identify the report as a systematic review.
Checklist response: The title of the article identifies the study as a meta-analytic synthesis.

Introduction rationale: Describe the rationale for the review in the context of existing knowledge.
Checklist response: Addressed in the introduction.

Introduction objectives: Provide an explicit statement of the objective(s) or question(s) the review addresses.
Checklist response: Addressed in the introduction.

Methods eligibility criteria: Specify the inclusion and exclusion criteria for the review and how studies were grouped for the syntheses.
Checklist response: Included in the methods and supplemental information.

Methods information sources: Specify all databases, registers, websites, organizations, reference lists and other sources searched or consulted to identify studies.
Checklist response: Included in the supplemental information.

Methods search strategy: Present the full search strategies for all databases, registers and websites, including any filters and limits used.
Checklist response: Included in the supplemental information.

Methods selection process: Specify the methods used to decide whether a study met the inclusion criteria of the review, including how many reviewers screened each record and each

report retrieved, whether they worked independently, and if applicable, details of automation tools used in the process.
Checklist response: Included in the supplemental information. A single reviewer screened records.

Methods data collection process: Specify the methods used to collect data from reports, including how many reviewers collected data from each report, whether they worked independently, any processes for obtaining or confirming data from study investigators, and if applicable, details of automation tools used in the process.
Checklist response: Included in the supplemental information. A single reviewer extracted information from publications.

Methods – data items: List and define all outcomes for which data were sought. Specify whether all results that were compatible with each outcome domain in each study were sought (e.g. for all measures, time points, analyses), and if not, the methods used to decide which results to collect. List and define all other variables for which data were sought (e.g. participant and intervention characteristics, funding sources). Describe any assumptions made about any missing or unclear information.
Checklist response: Included in the supplemental information.

Methods study risk of bias assessment: Specify the methods used to assess risk of bias in the included studies, including details of the tool(s) used, how many reviewers assessed each study and whether they worked independently, and if applicable, details of automation tools used in the process.
Checklist response: The methods section includes a component on the assessment of publication bias.

Methods effect measures: Specify for each outcome the effect measure(s) (e.g. risk ratio, mean difference) used in the synthesis or presentation of results.
Checklist response: Covered in the methods section.

Methods synthesis methods: Describe the processes used to decide which studies were eligible for each synthesis. Describe any methods required to prepare the data for presentation or synthesis, such as handling of missing summary statistics, or data conversions. Describe any methods used to tabulate or visually display results of individual studies and syntheses. Describe any methods used to synthesize results and provide a rationale for the choice(s). If meta-analysis was performed, describe the model(s), method(s) to identify the presence and extent of statistical heterogeneity, and software package(s) used. Describe any methods used to explore possible causes of heterogeneity among study results (e.g. subgroup analysis, meta-regression). Describe any sensitivity analyses conducted to assess robustness of the synthesized results.
Checklist response: Covered in the methods section and supplemental information.

Methods reporting bias assessment: Describe any methods used to assess risk of bias due to missing results in a synthesis (arising from reporting biases).
Checklist response: Covered in the methods section.

Methods certainty assessment: Describe any methods used to assess certainty (or confidence) in the body of evidence for an outcome.
Checklist response: Covered in the methods section and supplemental information.

Results study selection: Describe the results of the search and selection process, from the number of records identified in the search to the number of studies included in the review, ideally using a flow diagram. Cite studies that might appear to meet the inclusion criteria, but which were excluded, and explain why they were excluded.
Checklist response: Covered in the methods section and supplemental information.

Results study characteristics: Cite each included study and present its characteristics.
Checklist response: Covered in the supplemental information and online assembled database (data availability section of the article).

Results risk of bias in studies: Present assessments of risk of bias for each included study.
Checklist response: Risk of bias of individual retained studies was explored through a candidate predictor of study category.

Results of individual studies: For all outcomes, present, for each study: (a) summary statistics for each group (where appropriate) and (b) an effect estimate and its precision (e.g. confidence/credible interval), ideally using structured tables or plots.
Checklist response: Included in the Results section and supplemental information.

Results of syntheses: For each synthesis, briefly summarize the characteristics and risk of bias among contributing studies. Present results of all statistical syntheses conducted. If meta-analysis was done, present for each the summary estimate and its precision (e.g. confidence/credible interval) and measures of statistical heterogeneity. If comparing groups, describe the direction of the effect. Present results of all investigations of possible causes of heterogeneity among study results. Present results of all sensitivity analyses conducted to assess the robustness of the synthesized results.
Checklist response: Included in the Results section and supplemental information.

Results reporting biases: Present assessments of risk of bias due to missing results (arising from reporting biases) for each synthesis assessed.
Checklist response: Included in the Results section and supplemental information.

Results certainty of evidence: Present assessments of certainty (or confidence) in the body of evidence for each outcome assessed.
Checklist response: Included in the Results section and supplemental information.

Discussion: Provide a general interpretation of the results in the context of other evidence. Discuss any limitations of the evidence included in the review. Discuss any limitations of the review processes used. Discuss implications of the results for practice, policy, and future research.
Checklist response: Covered in the discussion section and supplemental information.

Registration and protocol: Provide registration information for the review, including register name and registration number, or state that the review was not registered.
Checklist response: The review was not registered.

Support: Describe sources of financial or non-financial support for the review, and the role of the funders or sponsors in the review.
Checklist response: Disclosed in the article.

Competing interests: Declare any competing interests of review authors.

Checklist response: Disclosed in the article.

Availability of data, code and other materials: Report which of the following are publicly available and where they can be found: template data collection forms; data extracted from included studies; data used for all analyses; analytic code; any other materials used in the review.
Checklist response: The data availability statement provides directions to access the database assembled for the meta-synthesis.

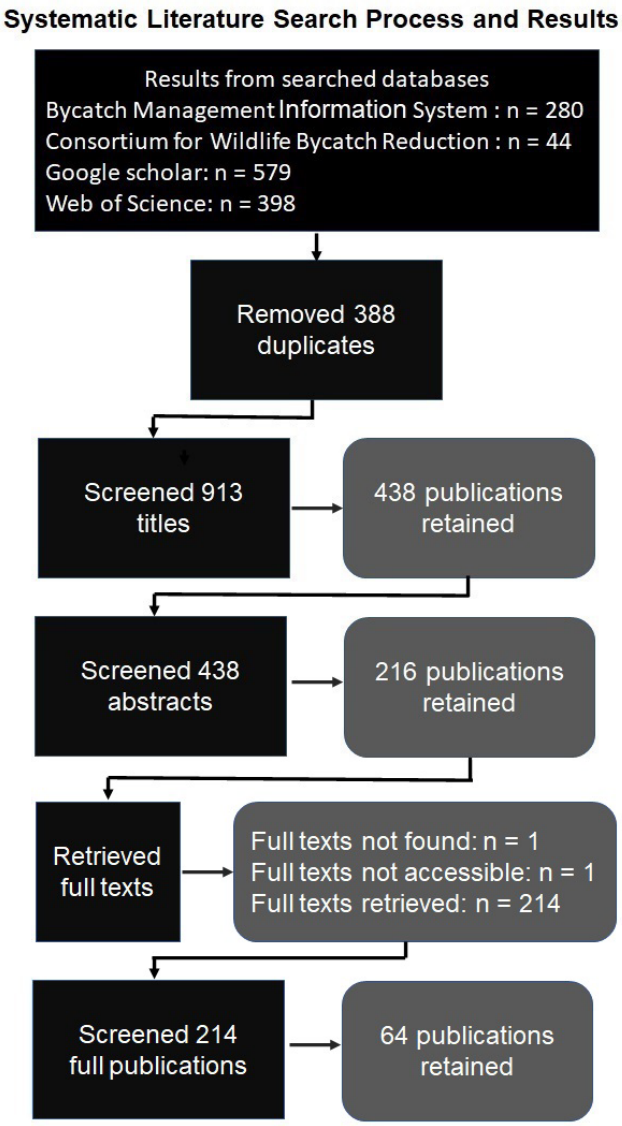

Fig. S5. Process and results of a systematic literature search conducted to compile publications on methods to mitigate bycatch and mortality of vulnerable species in pelagic longline fisheries (adapted from Haddaway et al. [46]).

S3.2. Elasmobranch Phylogenetic Structure

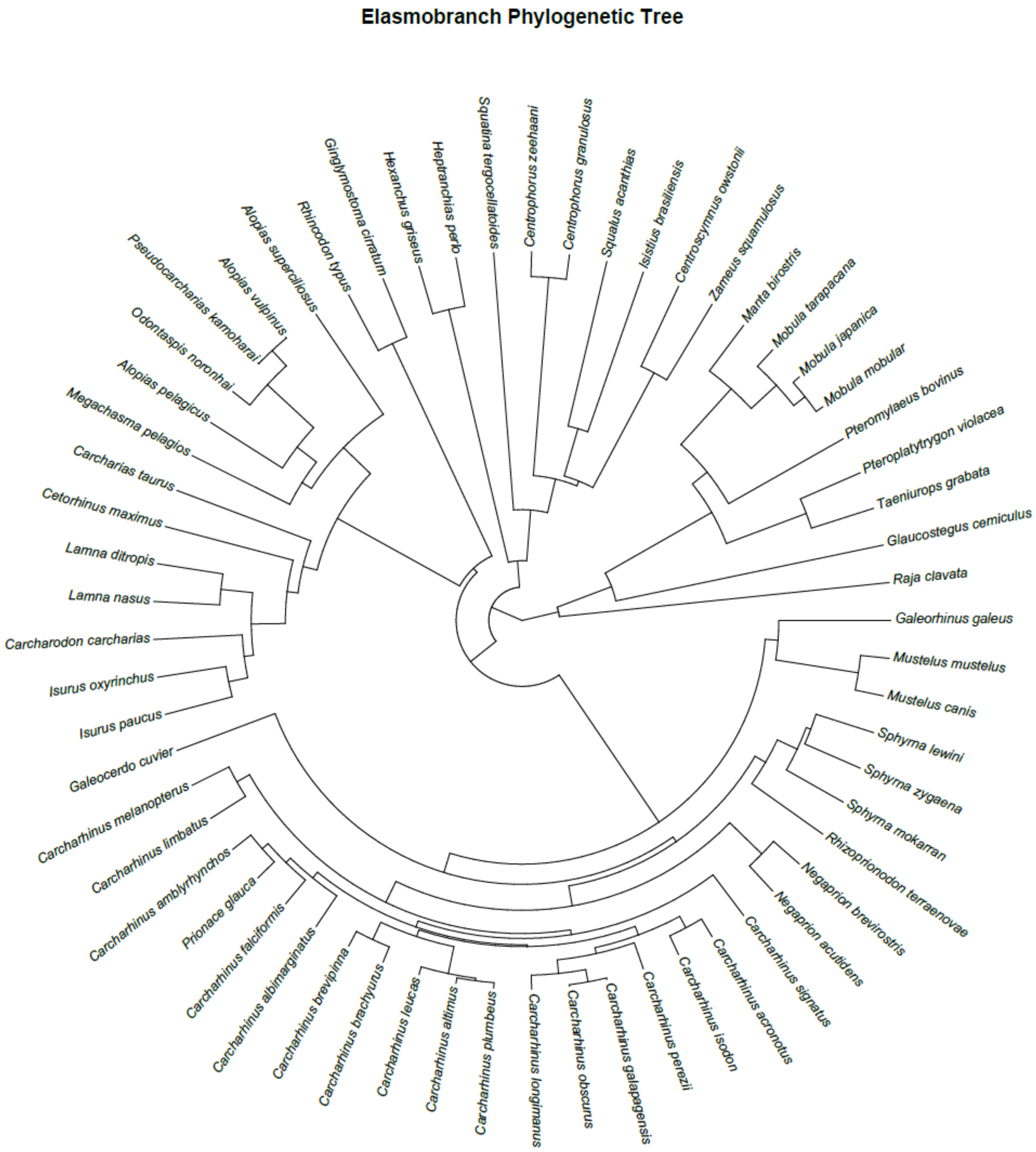

Fig. S6. Radial or circular tree plot showing the elasmobranch phylogenetic structure used for deriving the correlation matrices for the clade-specific Bayesian GAMMs. The phylogenetic tree was sourced online from https://vertlife.org/phylosubsets based on the phylogeny for *ca.* 1,200 Chondrichthyan species presented in [54].

S4. METHODS TO AVOID AND REDUCE ELASMOBRANCH CATCH IN PELAGIC LONGLINE FISHERIES

Methods to avoid and minimize shark and ray catch in pelagic longline fisheries can either (1) reduce effort; (2) cap bycatch or employ other output controls that incentivize the use of methods that reduce catch rates; or reduce one or more capture susceptibility attributes of: (3) areal and temporal overlap, (4) vertical overlap, and (5) selectivity. Examples of each category of approach follow.

- **Input controls on effort**. This includes, for example, limits on: the number of vessels (limited entry), vessel size, amount of gear, fishing days, number of sets, hooks per set, and soak duration [38,55].
- **Output controls**: Bycatch quotas result in the cessation of effort once the individual- or fleet-based threshold is reached. Retention bans (by species, sex, size), international trade bans, banning shark finning, and retention limits (individual- or fleet-based, for marketable species) do not directly affect catch levels, but might indirectly lead to fishers' use of methods that reduce catch rates.
- **Static and dynamic area-based management tools** to avoid or reduce areal or temporal overlap for spatially and temporally predictable bycatch hotspots. For instance, area-based approaches include: permanent static closures, such as longline closed areas near shallow submerged features; seasonal closures, such as at dynamic sites important for critical life history stages, including shark pupping and nursery areas; and quasi real-time measures, based on habitat suitability and species distribution models and fisheries-dependent observations implemented through move-on rules and voluntary industry fleet communication programs [56-58].
- Fishing methods that **reduce depth overlap** by adjusting the time of day of fishing and the fishing depth of the gear. For example, fishing depth can be managed by adjusting the number of hooks between floats (Section S2.3), length of mainline between floats, branchline and floatline lengths, distance between the floatline and adjacent branchline, and banning the use of 'shark lines' - branchlines that fish near the surface to target epipelagic sharks [15,59].
- **Increased selectivity** through fishing gear designs that (1) **Increase escapement** (e.g., weak leader material to enable some sharks to sever the line, [60]); (2) **Repel sharks** (e.g., electrical, chemical/olfactory, magnetic and rare earth electropositive metals, [61-62]; (3) **Create a mismatch with morphological characteristics** (e.g., hook shape and size – Section S2.2; [63-64]); and (4) **Reduce attractiveness** (e.g., bait species and artificial bait, not using light attractors; [65-66]).

S5. PRESCRIBED ELASMOBRANCH HANDLING AND RELEASE PRACTICES AND EQUIPMENT FOR PELAGIC LONGLINE FISHERIES

Based in part on findings on informative predictors of post-release mortality risk [5] (e.g., [67-68]), prescribed shark and ray handling and release methods for pelagic longline fisheries include [69-73]:

- Having a lifting device, bolt cutters, dehooker and line-cutters available where they are readily accessible by crew;
- Removing as much trailing line as safely possible;
- Removing hooks that are visible in the body or mouth;
- Using hooks and/or hook rings that degrade relatively quickly;
- Handling and releasing in the water, if possible, otherwise, bringing small elasmobranchs onboard but minimizing the duration out of the water;

- Minimizing the duration restrained, including by not using ‘lazy lines’ where crew attach catch to a line off the stern until the end of the haulback; and
- Various proscribed practices, including not: attempting to remove deeply ingested hooks, using lifting devices that risk entanglement, inserting anything into gill slits, putting tension on branchlines especially when deeply hooked, gaffing in the body, lifting by the head or tail, and putting excessive pressure on their bodies.

S6. REFERENCES

[1] Clarke, S., et al. 2014. Bycatch in Longline Fisheries for Tuna and Tuna-like Species: A Global Review of Status and Mitigation Measures. Food and Agriculture Organization of the United Nations, Rome.
[2] Stramma, L., et al. 2012. Expansion of oxygen minimum zones may reduce available habitat for tropical pelagic fishes. Nature Climate Change 2: 33–37.
[3] Ellis, J., et al. 2017. A review of capture and post-release mortality of elasmobranchs. Journal of Fish Biology 90: 653-722.
[4] Hyatt, M.W.; Anderson, P.A., O'Donnell, P. 2018. Influence of temperature, salinity, and dissolved oxygen on the stress response of bull (*Carcharhinus leucas*) and bonnethead (*Sphyrna tiburo*) sharks after capture and handling. Journal of Coastal Research 34: 818-827.
[5] Musyl, M., Gilman, E. 2019. Meta-analysis of post-release fishing mortality in apex predatory pelagic sharks and white marlin. Fish and Fisheries 20: 466-500.
[6] Pfaller, J., Chaloupka, M., Bolten, A., Bjorndal, K. 2018. Phylogeny, biogeography and methodology: a meta-analytic perspective on heterogeneity in adult marine turtle survival rates. Scientific Reports 8: 5852.
[7] Coelho, R., Fernandez-Carvalho, J., Lino, P., Santos, M. 2012. An overview of the hooking mortality of elasmobranchs caught in a swordfish pelagic longline fishery in the Atlantic Ocean. Aquat. Living Resour. 25: 311-319.
[8] Dapp, D., et al. 2017. Using logbook data to determine the immediate mortality of blue sharks (*Prionace glauca*) and tiger sharks (*Galeocerdo cuvier*) caught in the commercial U.S. pelagic longline fishery. Fishery Bulletin 115: 27-41.
[9] Gallagher, A., Orbesen, E., Hammerschlag, N., Serafy, J. 2014. Vulnerability of oceanic sharks as pelagic longline bycatch. Global Ecology and Conservation 1: 50-59.
[10] Schlaff, A., Heupel, M., Simpfendorfer, C. 2014. Influence of environmental factors on shark and ray movement, behaviour and habitat use: A review. Reviews in Fish Biology and Fisheries 24: 1089-1103.
[11] Massey, Y., Sabarros, P., Rabearisoa, N., Bach, P. 2019. Drivers of at-haulback mortality of sharks caught during pelagic longline fishing experiments. IOTC-2019-WPEB15-14_Rev1. Indian Ocean Tuna Commission, Mahe, Seychelles.
[12] Musyl, M., Gilman, E. 2018. Post-release fishing mortality of blue (*Prionace glauca*) and silky shark (C*archarhinus falciformes*) from a Palauan-based commercial longline fishery. Rev Fish Biol Fisheries 28: 567-586.
[13] Ochi, D., Ueno, S., Okamoto, K. 2021. Assessment of the effect of hook shape on fishing mortality of multi-taxa fish species using experimental longline operation data. Collect Vol Sci Pap ICCAT 78: 105-117.
[14] Mucientes, G., et al. 2009. Sexual segregation of pelagic sharks and the potential threat from fisheries. Biology Letters 5: 156-159.
[15] Musyl, M., et al. 2011. Postrelease survival, vertical and horizontal movements, and thermal habitats of five species of pelagic sharks in the central Pacific Ocean. Fisheries Bulletin 109: 341-368.
[16] Butcher, P., Peddemors, V., Mandelman, J., McGrath, S., Cullis, B. 2015. At-vessel mortality and blood biochemical status of elasmobranchs caught in an Australian commercial longline fishery. Global Ecology and Conservation 3: 878–889.
[17] Saidi, B., et al. 2019. Shark pelagic longline fishery in the Gulf of Gabes: Inter-decadal inspection reveals management needs. Mediterranean Marine Science 0: 532-541.
[18] Musyl, M., Moyes, C., Brill, R., Fragoso, N. 2009. Factors influencing mortality estimates in post-release survival studies: Comment on Campana et al. (2009). Mar Ecol Prog Ser 396: 157–159.
[19] Nunes, D., et al. 2019. Survivorship of species caught in a longline tuna fishery in the western equatorial Atlantic Ocean. Latin American Journal of Aquatic Research 47: 798-807.
[20] Coelho, R., Lino, P., Santos, M. 2011. *At-haulback Mortality of Elasmobranchs Caught on the Portuguese Longline Swordfish Fishery in the Indian Ocean*. IOTC-2011-WPEB07-31. Indian Ocean Tuna Commission, Mahe, Seychelles.
[21] Afonso, A., Santiago, R., Hazin, H., Hazin, F. 2012. Shark bycatch and mortality and hook bite-offs in pelagic longlines: Interactions between hook types and leader materials. Fisheries Research 131-133: 9-14.
[22] Gilman, E., Chaloupka, M., Swimmer, Y., Piovano, S. 2016. A cross-taxa assessment of pelagic longline bycatch mitigation measures: conflicts and mutual benefits to elasmobranchs. Fish and Fisheries 17: 748-784.
[23] Amorim, S., Santos, M., Coelho, R., Fernandez-Carvalho, J. 2014. Effects of 17/0 circle hooks and bait on fish catches in a southern Atlantic swordfish longline fishery. Aquatic Conservation: Marine and Freshwater Ecosystems 25: 518-533.

[24] Epperly, S., Watson, J., Foster, D. Shah, A. 2012. Anatomical hooking location and condition of animals captured with pelagic longlines: the grand banks experiments 2002-2003. Bulletin of Marine Science 88: 513-527.
[25] Watson, J., Epperly, S., Shah, A., Foster, D. 2005. Fishing methods to reduce sea turtle mortality associated with pelagic longlines. Can. J. Fish. Aquat. Sci. 62: 965-981.
[26] Curran, D., Beverly, S. 2012. Effects of 16/0 circle hooks on pelagic fish catches in three South Pacific albacore longline fisheries. Bulletin of Marine Science 88: 485-497.
[27] Serafy, J., et al. 2012. Circle hooks in commercial, recreational, and artisanal fisheries: research status and needs for improved conservation and management. Bulletin of Marine Science 88: 371-391.
[28] Campana, S., Joyce, W. Manning, M. 2009. Bycatch and discard mortality in commercially caught blue sharks *Prionace glauca* assessed using archival satellite pop-up tags. Marine Ecology Progress Series 387: 241-253.
[29] Pacheco, J., et al. 2011. A comparison of circle hook and J hook performance in a western equatorial Atlantic Ocean pelagic longline fishery. Fisheries Research 107: 39-45.
[30] Piovano, S., Clo, S., Giacoma, C. 2010. Reducing longline bycatch: The larger the hook, the fewer the stingrays. Biological Conservation 143: 261-264.
[31] Mas, F., Forselledo, R., Domingo, A. 2015. Mobulid ray by-catch in longline fisheries in the south-western Atlantic Ocean. Marine and Freshwater Research 66: 767-777.
[32] Smith, M., Warmolts, D., Thoney, D., Hueter, R. (Eds.). 2004. Elasmobranch Husbandry Manual. Captive Care of Sharks, Rays and their Relatives. Ohio Biological Survey, Columbus.
[33] Ward, P., Myers, R. 2005. Inferring the depth distribution of catchability for pelagic fishes and correcting for variations in the depth of longline fishing gear. Can. J. Fish. Aquat. Sci. 62: 1130-1142.
[34] Bigelow, K., Musyl, M., Poisson, F., Kleiber, P. 2006. Pelagic longline gear depth and shoaling. Fisheries Research 77: 173-183.
[35] Rice, P., Goodyear, C., Prince, E., Snodgrass, D., Serafy, J. 2007. Use of catenary geometry to estimate hook depth during near-surface pelagic longline fishing: Theory versus practice. North American Journal of Fisheries Management 27: 1148-1161.
[36] Moyes, C., Fragoso, N., Brill, R., Musyl, M. 2006. Predicting postrelease survival in large pelagic fish. Trans Am Fish Soc 135: 1389–1397.
[37] Gilman, E., et al. 2019. Robbing Peter to pay Paul: Replacing unintended cross-taxa conflicts with intentional tradeoffs by moving from piecemeal to integrated fisheries bycatch management. *Reviews in Fish Biology and Fisheries* 29: 93-123.
[38] Ward, P., Myers, R., Blanchard, W. 2004. Fish lost at sea: The effect of soak time on pelagic longline catch. Fisheries Bulletin 102: 179-195.
[39] Birkle, C., Pendlebury, D., Schnell, J., Adams, J. 2020. Web of Science as a data source for research on scientific and scholarly activity. Quantitative Science Studies 1:363–376.
[40] Bramer, W., Rethlefsen, M., Kleijnen, J., Franco, O. 2017. Optimal database combinations for literature searches in systematic reviews: A prospective exploratory study. Systematic Reviews 6: DOI 10.1186/s13643-017-0644-y.
[41] Haddaway, N., Macura, B., Whaley, P., Pullin, A. 2017. *ROSES for Systematic Review Protocols*. Version 1.0. DOI: 10.6084/m9.figshare.5897269.
[42] Haddaway, N., Macura, B., Whaley, P., Pullin, A. 2018. ROSES RepOrting standards for Systematic Evidence Syntheses: *pro forma*, flow-diagram and descriptive summary of the plan and conduct of environmental systematic reviews and systematic maps. *Environmental Evidence* 7: doi: 10.1186/s13750-018-0121-7.
[43] Pullin, A., Frampton,.G., Livoreil, B., Petrokofsky, G. (Eds). 2021. Section 3. Planning a CEE Evidence Synthesis. In Pullin A, Frampton G, Livoreil B, Petrokofsky G (Eds). *Guidelines and Standards for Evidence Synthesis in Environmental Management*. Version 5.0. Collaboration for Environmental Evidence.
[44] Sutton, A. 2009. Publication bias. In Cooper, H., Hedges, L., Valentine, J. (Eds.). *Handbook of Research Synthesis and Meta-Analysis*. 2nd Edition. Russell Sage Foundation, New York, pp. 435-452.
[45] Bayliss, H., Beyer, F. 2015. Information retrieval for ecological syntheses. Research Synthesis Methods 6:136-148.
[46] Haddaway, N., Macura, B., Whaley, P., Pullin, A. 2017. Additional File 3. ROSES RepOrting standards for Systematic Evidence Syntheses: *pro forma*, flow-diagram and descriptive summary of the plan and conduct of environmental systematic reviews and systematic maps. *Environmental Evidence* 7:7 doi.org/10.1186/s13750-018-0121-7.
[47] DFO. 2021. *Canadian Fisheries Observer Program. Unpublished Data for Atlantic Canadian Pelagic Longline Fisheries, 2001 to 2020*. Department of Fisheries and Oceans Canada, St. Andrews, New Brunswick.

[48] NMFS. 2021. *American Samoa Pelagic Longline Observer Program Database*. Pacific Islands Regional Office, National Marine Fisheries Service, Honolulu.
[49] NMFS. 2021. *Hawaii Pelagic Longline Observer Program Database*. Pacific Islands Regional Office, National Marine Fisheries Service, Honolulu.
[50] NMFS. 2021. *U.S. North Atlantic, Gulf of Mexico and Caribbean Pelagic Longline Observer Program Database*. Southeast Fisheries Science Center, National Marine Fisheries Service, Miami, Florida, USA.
[51] Afonso, A., Santiago, R., Hazin, H., Hazin, F. 2015. Corrigendum to "Shark bycatch and mortality and hook bite-offs in pelagic longlines: Interactions between hook types and leader materials". Fisheries Research 167: 278-279.
[52] Page M, et al. 2021. The PRISMA 2020 statement: An updated guideline for reporting systematic reviews. BMJ 372:doi.org/10.1136/bmj.n.71
[53] Page M, et al. 2021. PRISMA 2020 explanation and elaboration: Updated guidance and exemplars for reporting systematic reviews. BMJ 372:doi.org/10.1136/bmj.n.160
[54] Stein R, et al. 2018. Global priorities for conserving the evolutionary history of sharks, rays and chimaeras. Nature Ecology and Evolution 2: 288–298.
[55] Anderson, C., et al. 2018. How commercial fishing effort is managed. Fish and Fisheries 20: 268-285.
[56] Hazen, E., 2018. A dynamic ocean management tool to reduce bycatch and support sustainable fisheries. Science Advances 4: DOI: 10.1126/sciadv.aar3001
[57] Maxwell, S., et al. 2020. Mobile protected areas for biodiversity on the high seas. Science 367: 252-254.
[58] Hilborn, R., et al. 2021. Area-based management of blue water fisheries: current knowledge and research needs. Fish and Fisheries 23: 492-518.
[59] Hall, M., et al. 2017. Mitigating bycatch in tuna fisheries. Reviews in Fish Biology and Fisheries 27: 881-908.
[60] Ward, P., Lawrence, E., Darbyshire, R., Hindmarsh, S. 2008. Large-scale experiment shows that nylon leaders reduce shark bycatch and benefit pelagic longline fishers. Fisheries Research 90: 100-108.
[61] Hart, N., Collin, S. 2014. Sharks senses and shark repellents. Integrative Zoology 10: 38-64.
[62] Mitchell, J., McLean, D., Collins, S., Langlois, T. 2018. Shark depredation in commercial and recreational fisheries. Reviews in Fish Biology and Fisheries 28: 715-748.
[63] Gilman, E., Chaloupka, M., Musyl, M. 2018. Effects of pelagic longline hook size on species- and size-selectivity and survival. Reviews in Fish Biology and Fisheries 28: 417-433.
[64] Reinhardt J, Weaver J, Latham P et al. 2018. Catch rate and at-vessel mortality of circle hooks versus J-hooks in pelagic longline fisheries: a global meta-analysis. Fish Fish 19:413–430.
[65] Poisson, F., et al. 2016. Technical mitigation measures for sharks and rays in fisheries for tuna and tuna-like species: Turning possibility into reality. Aquat. Living Resour. 29: 402.
[66] Gilman, E., et al. 2020. Effect of pelagic longline bait type on species selectivity: A global synthesis of evidence. *Reviews in Fish Biology and Fisheries* 30: 535-551
[67] McGrath, S., Butcher, P., Broadhurst, M., Cairns, S. 2011. Reviewing hook degradation to promote ejection after ingestion by marine fish. Marine and Freshwater Research 62: 1237.
[68] FAO. 2019. *Joint Analysis of Shark Post-Release Mortality Tagging Results. Workshop Proceedings*. Areas Beyond National Jurisdiction Program, Food and Agriculture Organization of the United Nations, Rome.
[69] SPC, Resources Legacy Fund, Luen Thai Fishing Venture. 2012. Methods for longline fishers to safely handle and release unwanted sharks and rays. English and Chinese. https://www.bmis-bycatch.org/references/l7eksjd6. Pacific Community, Noumea, New Caledonia; Resources Legacy Fund, San Francisco; Luen Thai Fishing Venture, Shezhen, China.
[70] WCPFC. 2017. Best Handling Practices for the Safe Release of Mantas and Mobulids. Western and Central Pacific Fisheries Commission, Kolonia, Federated States of Micronesia.
[71] WCPFC. 2018. Best Handling Practices for the Safe Release of Sharks (Other than Whale Sharks and Mantas/Mobulids). Western and Central Pacific Fisheries Commission, Kolonia, Federated States of Micronesia.
[72] IOTC. 2020. Best Practice Handling and Release Guidelines for Mobulid Rays Caught in IOTC Fisheries. Version 6. Indian Ocean Tuna Commission, Mahe, Seychelles.
[73] Jorgensen, S., et al. 2022. Emergent research and priorities for elasmobranch conservation. Endangered Species Research 47: 171-203
[74] Dolce J, Wilga, C. 2013. Evolutionary and ecological relationships of gill slit morphology in extant sharks. Bulletin of the Museum of Comparative Zoology 161: 79-109
[75] Higgins, J., Thompson, S. 2002. Quantifying heterogeneity in a meta-analysis. Statistics in Medicine 21: 1539–1558.
